# Users’ polarisation in dynamic discussion networks: The case of refugee crisis in Sweden

**DOI:** 10.1371/journal.pone.0262992

**Published:** 2022-02-09

**Authors:** Elizaveta Kopacheva, Victoria Yantseva

**Affiliations:** 1 Department of Political Science & Centre for Data Intensive Sciences and Applications (DISA), Linnaeus University, Växjö, Sweden; 2 Department of Social Studies & Centre for Data Intensive Sciences and Applications (DISA), Linnaeus University, Växjö, Sweden; University of Oxford, UNITED KINGDOM

## Abstract

This paper presents a study on the dynamics of sentiment polarisation in the active online discussion communities formed around a controversial topic—immigration. Using a collection of tweets in the Swedish language from 2012 to 2019, we track the development of the communities and their sentiment polarisation trajectories over time and in the context of an exogenous shock represented by the European refugee crisis in 2015. To achieve the goal of the study, we apply methods of network and sentiment analysis to map users’ interactions in the network communities and quantify users’ sentiment polarities. The results of the analysis give little evidence for users’ polarisation in the network and its communities, as well as suggest that the crisis had a limited effect on the polarisation dynamics on this social media platform. Yet, we notice a shift towards more negative tonality of users’ sentiments after the crisis and discuss possible explanations for the above-mentioned observations.

## Introduction

With the rapid development of social media, modern societies are facing new challenges of the digital age. While right-wing populism is on the rise in Europe [1], social networking sites represent communication platforms that are being used by a variety of social forces for deliberation, persuasion and recruitment of new members. Likewise, social media are praised for enabling grass-roots activism [2] and for allowing users’ exposure to cross-ideological content [3]. At the same time, they have also been found to trigger group polarisation and the emergence of echo chambers [4–6].

The phenomenon of users’ polarisation has been reported in a large number of studies (e.g., [4, 5, 7–10]), however, up to date, this phenomenon has mostly been studied in the context of political ideologies and orientations (e.g., [11, 12]). Despite that the researchers have collected sufficient evidence for the particular conditions that cause polarisation among the social media users (see Related work section), we suggest that there is a need to go beyond the issue of social media users’ polarisation based on political party support or membership. In order to provide a more complex and nuanced picture of polarisation, it is essential to address a wider range of socially relevant issues, one example of which is the immigration topic. It can be described as a controversial and pressing issue for many Western democracies, especially in the light of the recent European refugee crisis, growing support for right-wing parties [13, 14] and growing hostility towards newcomers [15], as well as the securitisation of the immigration policies [16, 17].

Despite the existing evidence that, in some instances, online networks can create communities where heterogeneous discussions are more common than homogeneous ones [18, 19], many scholars suggest that social media-driven networks tend to expose users to the content that supports their already existing attitudes and create echo-chamber-like environments [20–22]. In light of these findings, it is evident that social media can serve as echo-chambers for migrant-hostile users. Earlier, it has been suggested that social media platforms assist right-wing populist movements in community fragmentation [23]. It has been found that right-wing activists use social media not only for information dissemination but also for the recruitment of new members and anti-immigrant mobilisation [24, 25]. Therefore, given the widespread use of social media by various political movements, it is necessary to explain the mechanisms behind the growth of right-wing populism and group polarisation in social media.

Our empirical case for the analysis in this paper is a social network of the Swedish-speaking Twitter users who discussed immigration in 2012–2019. In our study, we concentrate on the role of the European refugee crisis in 2015, which has been argued to cause anti-refugee mobilisation on the social networking platforms [26]. In 2015, Sweden experienced an unprecedented inflow of asylum seekers and accepted the second highest number of refugees per capita in the European Union [27]. These events, however, resulted in the government’s recognition of failure to accommodate such a large number of people and in the adjustment of the asylum legislation in accordance with the EU’s minimum level [28]. Some scholars characterised this crisis as an exogenous shock for the Swedish society [29, 30]. Since the existing evidence about the effects of political crises and other disruptive events on the dynamics of group polarisation is limited (with the exceptions of [31–33]), we seek to contribute to the existing research by closing the gap in this area of knowledge.

The refugee crisis of 2015 has certainly left its mark on the political discourse not only in Sweden, but also in other European countries, and has shaped the agenda of both mainstream and participatory media. Some scholars suggest that the crisis had effects on the European immigration policies, as well as on public opinion and attitudes to both forced and voluntary migration. The crisis, it has been argued, caused the mainstream media perceptions of migrants and refugees to change towards more prejudiced and hostile ones [34], while the attitudes of the Europeans holding right- or left-wing views have become more polarised [35]. Representing a “hybrid media environment”, social media have been found to be suitable platforms for polarisation among those who used online social networks to discuss the events of 2015 [36]. It has also been suggested that the users’ sentiments during the crisis were mostly negative [37, 38].

To sum up, our ambition is to contribute to the study of group polarisation dynamics and selective exposure in online networks. In this paper, we focus on the immigration topic as a potentially polarising issue and on the European refugee crisis as a disruptive event that could intervene with the overall polarisation dynamics in online discussions. Since there is a lack of empirical evidence on the effects of political crises on the dynamics of polarisation in social media, we expect that the events of 2015 triggered a growing interest in the issue of migration (thus, that 2015 is associated with an increased inflow of users to the social media communities). We hypothesise that 2015 is linked to an increase in sentiment polarisation (as proposed by [32, 33]). In addition to that, we expect to detect the prevalence of homogeneous relationships in the network communities (as suggested by [20–22]) and persistent changes in the levels of polarisation (in relation to the findings of [31]).

In the study, we use Twitter messages that were posted between 2012 and 2019 (1 198 985 tweets in total) and that discuss the issue of immigration in the Swedish language. In our analysis, we focus only on those users who were persistently active in the discussions over time. Indeed, there is an ongoing discussion in the political participation research if political activism in social media (often referred to as “slacktivism” [39, 40]) can be considered a type of political participation. It has been suggested that the majority of the social media users play the role of content-consumers rather than content-creators (i.e., active users) [41]. Some studies admit that social or/and political activism implies active engagement in discussions with others [42, 43]. Moreover, Rojas’s study on the correlation between active political discussions and political participation provides evidence for the fact that the frequency of political discussions is positively correlated with both civic and institutional types of engagement [44]. In a study of users’ polarisation, it seems important to distinguish between content-consumers and content-creators. Active users have the power to engage new users into political discussions and can potentially mobilise people into political action (e.g., protesting, signing petitions, etc.). Thus, polarisation in those groups can be the most dangerous with respect to radicalisation and the growth of right-wing populism [6, 45].

## Related work

### Group polarisation

The empirical evidence for group polarisation has been obtained as early as 1969 [46] and further corroborated in the 70-s [47, 48]. In its essence, the theory of group polarisation suggests that the key underlying condition for polarisation to occur is the members’ interactions within the group. In “The Law of Group polarisation”, Cass Sunstein [49] argues that the final point of the group discussion would gravitate towards a more extreme opinion [49, p. 9]. The scholar highlights two factors, i.e., opinion homogeneity and the mechanism of social influence, which affect the group’s movement towards the extremity. The former (opinion homogeneity) can be described as the “limited argument pools” available to the group members [49, p. 4], whereas the mechanism of social influence refers to the agents’ inclination to adopt other group members’ attitudes and behaviours [49, p. 4].

Interestingly enough, Sunstein also suggests that polarisation is especially likely if the group can position itself against another outgroup [49, p. 21]. This statement is extremely relevant for the studies of the polarisation dynamics in the immigration context since migrants and refugees (or, alternatively, those supporting current immigration policies) can be easily represented as “The Other”. Another case when groups are especially likely to polarise is when they already hold extreme opinions on a given topic. Since social media have been actively exploited by the right-wing movements to push forward the migrant-hostile agenda, we can expect that longer involvement in the right-wing group discussions results in the further radicalisation of the group members. Sunstein referred to this phenomenon as to the “individual polarisation toward within-group extremes” [49, p. 11]. In addition to that, anonymity, one of the distinctive features of online communication, can serve as a breeding ground for polarising deliberations that causes people to adopt extreme opinions [49, p. 23].

### Selective exposure and opinion homophily

With the onset of the World Wide Web era, the phenomenon of polarisation in online settings has been reported in a large number of studies (e.g., [4, 5, 7–10]). This finding has been coupled with an observation that, in online communities, users tend to be exposed to the opinions that already correspond with their own and to connect to the users with similar views. This phenomenon has been referred to as a mechanism of selective exposure [3, 7, 50]. Another important finding is a mechanism of confirmation bias that makes users choose and favour the information that confirms and supports their existing beliefs [51–54]. On top of this, it has been proposed that the algorithmic architecture of online networking platforms fuels these tendencies by suggesting content that meets users’ pre-existing beliefs, thereby fostering biased perceptions of the topics in question [55–57]. However, this filter bubble hypothesis has lately been refuted in a number of studies (see, for instance, [58]).

In contrast to Sunstein’s statement about the role of social homogeneity in group polarisation, a range of studies have posited that social media frequently expose users to alternative views (so-called “cross-ideological exposure” [59–62]). Researchers have also pointed at the co-existence of multiple communities in online networks with varying degrees of polarisation with regard to the topic [63] and at the fact that the processes of polarisation and cross-ideological exposure can simultaneously exist in online networks [64, 65]. Despite the presence of homogeneous user groups within the networks, at least some of the communities can still be characterised as heterogeneous in terms of the users’ opinions and ideological dispositions. This mitigates the effects of selective exposure and has been named as “enclaves of exposure” [18] and “open forums” [19]. Yet, some of the studies have also provided evidence that cross-ideological exposure in online discussions can actually increase users’ polarisation [12].

### The dynamics of users’ polarisation

With regard to the dynamic aspect of user polarisation, the existing studies suggest that higher user involvement and longer discussions produce more negative attitudes towards the topic of discussion [6, 45]. Moreover, the empirical evidence supports the suggestion that polarisation increases with time [66]. The prevalence of negative emotions in the news context enables its rapid diffusion in online networks [67, 68], while users’ sentiment negativity, in general, creates a favourable environment for their polarisation [69]. Partisan users were found to be especially likely to engage in homogeneous connections and produce more polarised network structures [70]. However, cross-platform differences in the polarisation dynamics have also been mapped by the previous research. Thus, scholars found de-polarising dynamics of discussions on WhatsApp and Facebook [71, 72] and polarising effect of discussions on Twitter [72, 73].

In social sciences, group or opinion polarisation on social media has mainly been studied with the focus on users’ political orientations and partisanship (e.g., [5, 11, 74, 75]) while a far smaller number of studies have been dedicated to other socially relevant and controversial issues [76–78]. Due to the fact that political orientations inevitably influence people’s attitudes to socially relevant issues, such as immigration policies, climate change or nuclear power use, we find it relevant to suggest that the studies of users’ polarisation on social media can be extended beyond the political ideology issue. Such research can explore the case of users’ polarisation on the topic of immigration in Sweden, a complex phenomenon that cannot be explained solely by the users’ political orientations and attitudes.

At the same time, only a limited number of studies investigated the association between disruptive events or crises and the dynamics of opinion polarisation. For instance, it has been found that crises or political conflicts are linked to the growth of opinion polarisation in the discussion networks [32, 33] and that unexpected events and crises can result in long-term sentiment changes in them [31]. Still, some further evidence needs to be obtained. Because the refugee crisis represented a disruptive event that shaped the European public agenda in 2015 [29, 30], social scientists have argued that it provoked a heavy clash between a variety of discourses [79].

Throughout this paper, we use the notion of sentiment polarisation rather than opinion polarisation, although the concepts of opinion and sentiment are often used interchangeably. According to Merriam-Webster Online Dictionary, sentiment can be defined as “an attitude, thought, or judgment prompted by feeling” [80]. Sentiments, accordingly, are different from opinions in that they “are prompted by emotions” [81], yet, sentiments cannot be reduced to emotions since they are argued to be more conscious and stable over time [81]. To take into account this subjective component of users’ expressions on the topic seems to be a suitable approach to study the refugee crisis that, as suggested above, was perceived as an outstanding event. One more reason to use the notion of “sentiment polarisation” rather than “opinion polarisation” is a methodological one. In particular, opinions are more difficult to distinguish, whereas users’ sentiments can be classified with a wider range of analytical tools, which is especially relevant given that the Swedish language can be described as an under-resourced language.

In our definition of polarisation, we follow DiMaggio et al. who define it as “…a state and a process. Polarisation as a state refers to the extent to which opinions on an issue are opposed in relation to some theoretical maximum. Polarisation as a process refers to the increase in such opposition over time” [82, p. 693]. In this paper, we are concerned with the dispersion of sentiments and the bimodality of their distribution, both of which have been named as typical attributes of polarisation [82, p. 693], as well as with the changes of these attributes with time. In particular, these two properties allow measuring different dimensions of polarisation: whereas dispersion identifies how “far apart” or distant two opinions are from each other, the bi-modality of opinion or sentiment distribution quantifies how distinct or separated these groups or clusters of opinions are [82, p.694]. Thus, growing dispersion of sentiment values would denote stretching sentiment repertoire and growing distance between the users or groups expressing those sentiments (and, potentially, their growing extremity). A more pronounced bi-modality of sentiment distributions, in its turn, would mean a sharper distinction and isolation between the groups holding different sentiments.

### Hypotheses

Building our expectations on the results of the previous research outlined above, we expect that users’ communication patterns on Twitter can be characterised by:

(H1) an overall increase of sentiment polarisation in 2015 on the network and community levels,
(H2) the occurrence of persistent (rather than short-term) changes in the levels of polarisation in the network and its communities, and
(H3) the prevalence of sentiment and relationship homophily in the dynamic communities, which serves as a factor for the communities’ formation.

## Methods

### Data and information retrieval

Within the study, we applied dynamic network analysis and text analysis to examine 1 198 985 Twitter messages posted in the period between 1st of January 2012 and 31st of December 2019. The tweets were collected via the official Twitter full-archive search using the tools of *rtweet* package [83] in the R computing environment [84]. The query included the following terms: refugee(-s), migrant (-s), immigrant (-s), asylum seeker (-s), newcomers, immigration and migration. All tweets collected for the analysis were written in the Swedish language (please see S1 File for the Swedish query terms). All retweets were excluded from the initial search, since, in this study, we examined the reply network (where the link between user *A* and *B* represent a reply of user *A* to *B* or a mention of user *B* by *A*) to track the development of sentiment polarisation trajectories. Previously, retweet networks (where the link between user *A* and *B* represents a retweet posted by user *A*, or an original tweet written by user *B*) were used to examine the patterns of user influence [e.g., 85, 86], which is not the focus of our study.

**Fig 1:**
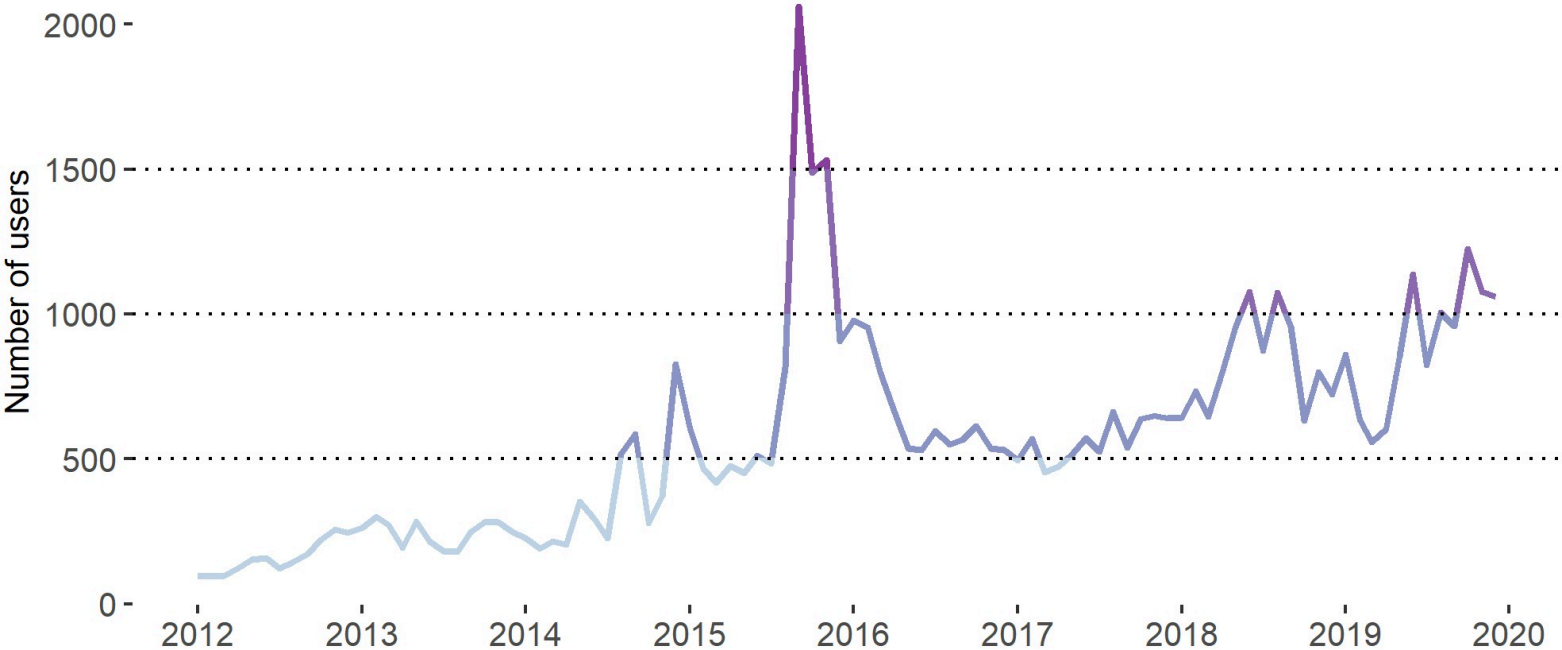
Number of content-creating users per month. *Notes*: The line is fitted into the points showing the number of users who posted at least one message in a given month. *Total N of users* = 9 300.

As the result of the data extraction, we received a network consisting of 9300 unique users, which could be either organisation or personal accounts. The users were tweeting on an infrequent basis. Fig 1 shows that 2015 is associated with the high level of the content-creators’ activity. In the meanwhile, prior to 2015, less than 500 of users posted messages each month. Moreover, almost 40% of the users involved into the discussions within the period of one month and only 1519 unique users tweeted about migration for at least 10 months.

**Fig 2:**
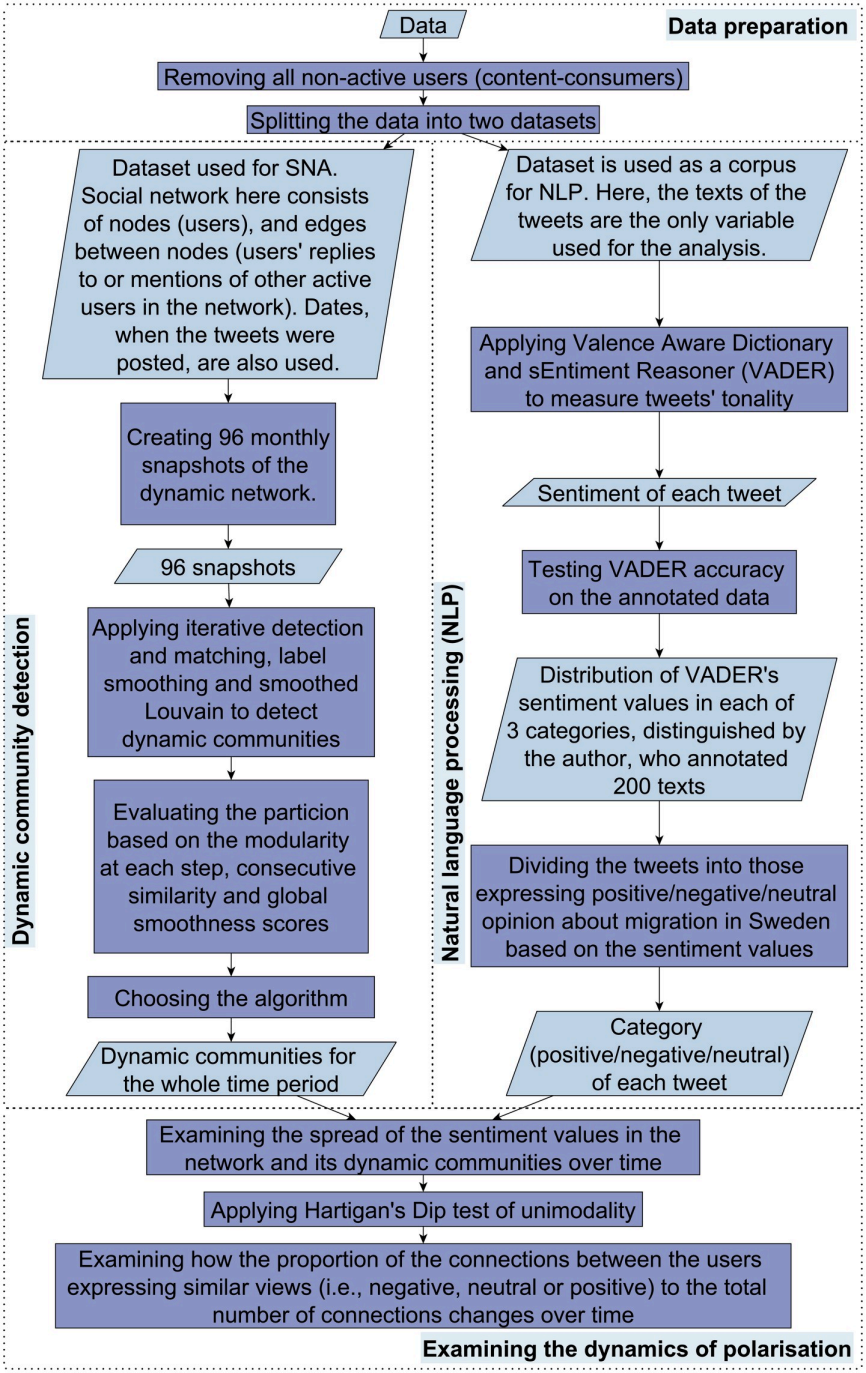
The step-wise analysis of the retrieved data.

We proceeded with the analysis as specified by Fig 2.

### Dynamic community detection

Due to the fact that social media present highly dynamic and constantly evolving communities that split, emerge, expand, contract and sometimes have short life cycles [87], we applied a dynamic community detection technique to distinguish communities persistent over time (see the part of Fig 2 titled “Dynamic community detection”). When studying polarisation, it is crucial to take into account the fact that members of long-living communities may change their sentiments over time. For example, it was found that longer discussions produce more negative attitudes towards the topic of discussion [6, 45]. Yet, there is a lack of research addressing the dynamic aspect of polarisation in online networks (see [88–90] for some exceptions). This gap may be explained by the limited number of tools available for the analysis of dynamic network communities and the restraints of those tools when it comes to working with big data (e.g., lack of RAM or HDD, slow processing speed).

In particular, the existing techniques utilised to detect dynamic communities may be divided into two groups: those that track (i.e., map) the evolution of communities in an “time-ordered sequence of static networks” [91] and those that gather information on the fly and add nodes into communities with each edge being created. The latter model-type has been titled “temporal networks” [92]. Despite the fact that temporal network mining is more often addressed by network analysts [93, 94], there are not many reliable techniques that assist in extracting communities in such complex networks. In the meanwhile, tracking the evolution of communities detected in static networks (i.e., snapshots of a dynamic network) is a more trivial task with a wide range of existing tools developed to facilitate the process (read more about the constraints of the dynamic community discovery via community evolution tracking in [95]). Still, it is worth mentioning certain limitations and assumptions of both community detection in static networks and community mapping approaches. Thus, the differences between community detection algorithms may concern the assumptions in regard to how many communities a user can belong to: some algorithms assume that a node can be in only one community at a time (in regard to detection of so-called crisp communities), while others suggest that a node can belong to several communities. For another thing, some community detection algorithms address the issue by finding groups of users (or nodes) densely connected to each other (i.e., modularity based approaches) while others may look at other properties of the nodes, e.g., group the nodes exhibiting similar connection patterns (i.e., approaches based on the stochastic block model framework) [95].

More assumptions are involved when trying to map the communities found at each snapshot. Thus, community mapping algorithms can be divided into 3 categories: those without any smoothness criteria, assuming that the communities found at timestamp *τ* depend only on the state of the network at that time-period; temporal trade-off algorithms, which look for optimal communities that depend on the typology of the network both at timestamp *τ* and previous timestamps and cross-time algorithms, which consider all of the timestamps to find an optimal solution [96]. When working with the social media data and trying to model dynamic events, temporal trade-off and cross-time approaches are the preferred solution as they aim to find communities coherent in time [95]. Still, many of those algorithms track the evolution of crisp communities (where a node can belong to only one community at at timestamp *τ*) [97], which can also, be seen as a limitation when working with the social media data, in particular.

Due to the specified methodological limitations, we extracted communities in 96 static networks, each representing users’ interactions in a given month, and tracked (i.e., mapped) the evolution of communities using and comparing the results of iterative detection and matching (i.e., a temporal trade-off approach) [98], label smoothing [99] and smoothed Louvain (i.e., cross-time approaches) [100]. Nodes (i.e., users) within each network were connected with the links representing users’ replies to or mentions of other active users. We decided to leave only active users who posted more than 5 messages in a month (none of which was a retweet). Users who wrote less than 5 messages in a month accounted for more than 90% of all users in our network, however, they mostly added noise to the data. If a user tweeted more than 5 messages one month but did not do so the next month, the user would be present only in the network of the time-period, in which this user posted more than 5 messages. In that way, we controlled for the growth of the network based on the length of the time-period examined. Thus, only the escalating popularity of Twitter as a network for political opinion expression and the growing interest in the topic of migration in Sweden account for the increasing number of users presented by the dynamic network.

Once constructing the dynamic network, we applied three community evolution algorithms to detect users’ communities persistent over time. The algorithms differ in terms of both community detection in each of the network snapshots (i.e., in each of 96 static networks) and the community evolution tracking procedure. In particular, we have evaluated smoothed Louvain [101] and Clauset-Newman-Moore greedy modularity maximisation [102] community detection methods, along with community matching in consecutive snapshots [based on the rules described in 97], label smoothing [99] and smoothed Louvain, where community detection takes place based on the partition on a previous time-step [100]. We evaluated dynamic partition based on the modularity at each step, consecutive similarity and global smoothness scores, such as the average value of partition smoothness, node smoothness and label smoothness [97]. As a result, the highest partition, node and label smoothness were reached by applying iterative community detection using Clauset-Newman-Moore greedy modularity maximisation and matching and smoothed Louvain. Thus, we proceeded with the analysis partitioning graph into the dynamic communities received after utilising iterative detection and matching. One of the limitations of using iterative community detection (i.e., a temporal trade-off approach) is that the density of the communities at each time-period is decreased to map communities coherent in time. Still, this is a limitation that characterises all trade-off and cross-time community mapping approaches [95].

### Natural language processing

In parallel with the dynamic community mining, we analysed the tonality of the tweets written by the content-creators in each of the communities (see the part of Fig 2 titled “Natural language processing”). To calculate sentiment values, we used a Swedish version of the Valence Aware Dictionary and sEntiment Reasoner (VADER) [103]. VADER is a tool that was specifically fine-tuned to measure sentiments in social media and has been found to outperform traditional lexicon-based tools for sentiment analysis [103]. The analytical tool allowed to measure both the polarity and intensity of sentiments. Thus, by applying VADER to the data, we received the tonality of the tweets on the scale from -1 (signifying the negative sentiment) to +1 (suggesting positive tonality).

**Fig 3:**
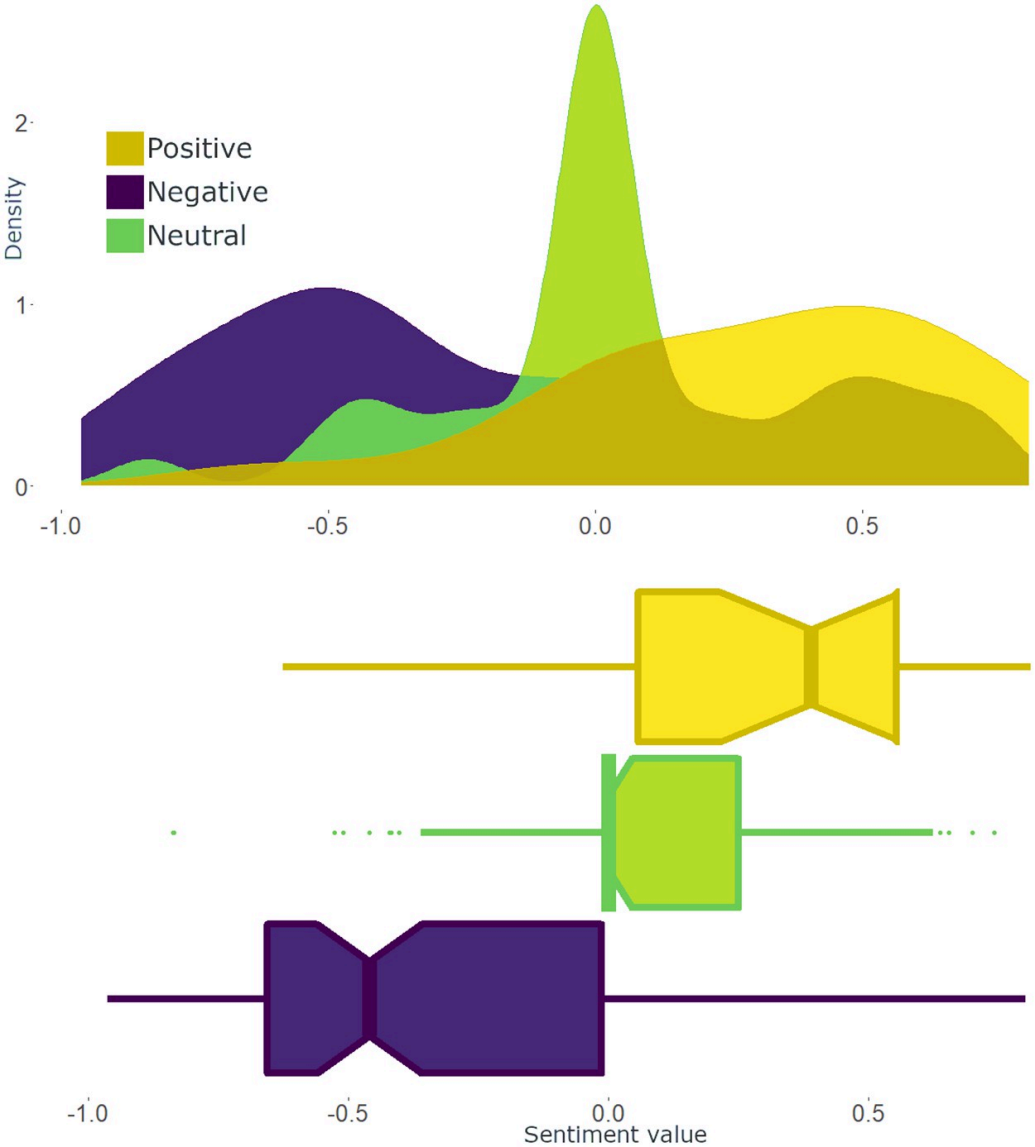
Test statistics on the VADER’s accuracy of measuring the tweet tonality. *Notes*: The top figure shows density (Y-axis) of the sentiment values (X-axis) assigned by VADER [103] to each annotated tweet. The colours represent the categories distinguished by the author who labeled the tweets. The bottom figure shows the overlap between the sentiment values of each stance group distinguished by the annotator. The boxplot also provides some summary statics on the distribution of the sentiment values (X-axis) assigned by VADER [103] in the stance categories found by the annotator. *N of tweets* = 200. *N of sentiment groups* = 3 (positive, neutral and negative).

Lexicon’s reliability was assessed using 200 tweets manually annotated by one of the authors (see the distribution of the sentiment values in the three categories in Fig 3). The results of the reliability test showed to be lower than the accuracy, precision and recall values for the English-language version of VADER applied to Twitter data, but, at the same time, on par with the classification results for other types of corpora (according to Hutto and Gilbert, three-class sentiment classification problems tend to yield the accuracy of around 60% [103]). In particular, the values of accuracy, precision and recall are 0.62, 0.6, 0.63 accordingly and the F1 score is 0.56. Moreover, we tested several preprocessing steps, in particular, lowercasing, stemming and removing punctuation, however, their use had no effect on the final classification accuracy of the labeled dataset.

Based on the distribution of VADER’s sentiment values in three categories of the manually annotated dataset, the categories of the rest of the tweets were assigned as follows: negative tweets are the messages with the sentiment values in the interval [-1; -0.092), positive tweets are those with the sentiment values in the interval (0.1284; 1] and neutral tweets are posts with the sentiment values in the interval [-0.092; 0.1284]. The initial border values were calculated using the estimated kernel cumulative distribution function (CDF), such as 1 − *CDF*_*neg*+*neu*_(*s*_1_) = *CDF*_*pos*_(*s*_1_) and 1 − *CDF*_*pos*+*neu*_(*s*_2_) = *CDF*_*neg*_(*s*_2_), where *CDF*_*pos*/*neu*/*neg*_ is the cumulative distribution function of sentiment values in the category positive/negative or neutral and *s*_1/2_ is the border sentiment value. After that, the grid search approach was applied to find the border values that maximise the accuracy of the model on the annotated data, the value of *y* = (*T*_*pos*_ − *F*_*pos*_) + (*T*_*neg*_ − *F*_*neg*_)(as suggested by [104]), where *T*_*pos*/*neg*_ are the true positive or negative and *F*_*pos*/*neg*_ are the false positive or negative values, and recall for all of the categories. The grid search was applied to the values in the interval [*s*_1_ − 0.3; *s*_1_ + 0.3] and [*s*_2_ − 0.3; *s*_2_ + 0.3] and the values closest to the initial (*s*_1_ and *s*_2_), yet, the ones that maximise accuracy, *y* = (*T*_*pos*_ − *F*_*pos*_) + (*T*_*neg*_ − *F*_*neg*_) and recall were chosen (see the S1 File for more details).

**Fig 4:**
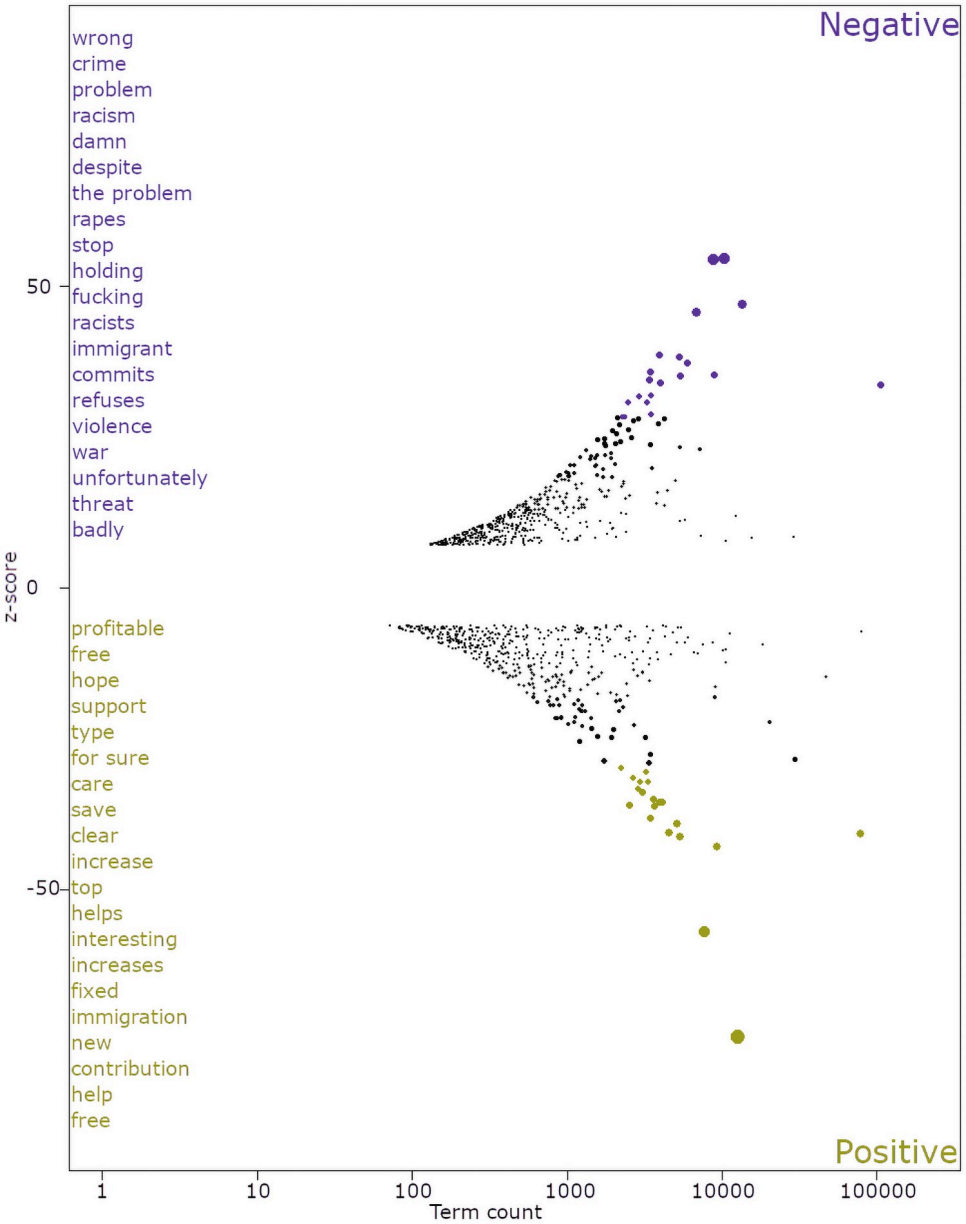
Top 20 words associated with the tweets with positive and negative tonality. *Notes*: Term-category association (TCA) [105] analysis was applied to identify the words associated with negative and positive tonality. The list of top 20 words was machine-translated. Please, find the list of top 20 words in the original language in S1 File. Y-axis shows z-scores for each of the identified terms. X-axis shows the term count. *N of tweets* = 678 677.

Moreover, the term-category association technique within the *SpeedReader* package [105] was also applied to check for reliability. That allowed distinguishing the words that were most likely to be associated with the groups of negative, positive and neutral tweets (see Fig 4). Those words showed to be consistent with the categories of the tweets.

### Examining the dynamics of polarisation

The received sentiment values allowed us to measure the dynamics of polarisation in both the dynamic communities and the network as a whole (see the part of Fig 2 titled “Examining the dynamics of polarisation”). In this paper, the dynamics of polarisation were assessed by studying the sentiment changes in each of the dynamic communities and in the network as a whole. When analysing polarisation in dynamic communities, we focused only on the main communities of users, i.e., communities in which users appeared most often (e.g., the main community of user *U*_*a*_ is the mode of the distribution of *U*_*a*_’s communities, where the set of *U*_*a*_’s communities consists of 96 values corresponding to 96 time-periods).

We tested for bimodality of the distributions of users’ sentiments using visual analytic tools and applying Hartigan’s Dip test of unimodality [106] on the sentiment values’ distributions across the study period. In particular, we inspected density of sentiment values at different time points (i.e., we studied the distribution of the sentiment values in January 2012, which is the first month of the examined period, in December 2015, which illustrates the distribution in the midst of the crisis, in October 2017, characterised by the significant changes in the sentiment value distribution, and in December 2019, which is the last month of the examined period). On top of this, we studied the changes in the mean and standard deviation of sentiments over time both in the whole network and in the dynamic communities.

Finally, we examined the nature of relationships between the users to distinguish if the latter tend to form homophilic or heterophilic relationships. We predict that sentiment homophily serves as a factor for community building in our discussion network. Thus, we analysed the proportion of connections between the users expressing similar views (i.e., negative—negative, neutral—neutral and positive—positive) to the number of all connections within the examined time point. The growing disproportion in the number of connections between the users expressing similar and opposite views indicated the tendency of users to form homophilic relationships.

### Additional details on the methods used in this study

All analyses were performed using the R 4.0.1 [84] and Python 3.9 [107] platforms as well as a number of additional packages mentioned earlier. One of the limitations of the study is the specific characteristics of the VADER tool, which was used to measure the tonality of the tweets. In particular, VADER is the model that was fine-tuned on English texts [103]. Inaccuracies in the machine-translation may have caused lower accuracy of the model. Despite that fact, compared with other techniques, tools and dictionaries, which were tested on the manually-labeled data, VADER showed higher accuracy, precision and recall. That may be partly explained by the fact that VADER was specifically fine-tuned to measure polarity and intensity of sentiment in social media. Moreover, the scope of the other existing sentiment dictionaries in Swedish is somewhat limited, which can explain their low performance on the annotated data.

For more details on the research-design see S1 File.

## Results

The removal of content-consuming users from the network allowed to reduce noise in the static networks and receive a smaller number of dense communities. The subsequent utilisation of community evolution tracking allowed us to distinguish 722 dynamic communities, with only 8 of them including more than 100 users. At the same time, at least 40% of users (3698 out of 9300) were not assigned to any of the dynamic communities (i.e., did not reply to or mention any of the active users and were not mentioned or replied to by other content-creators).

**Fig 5:**
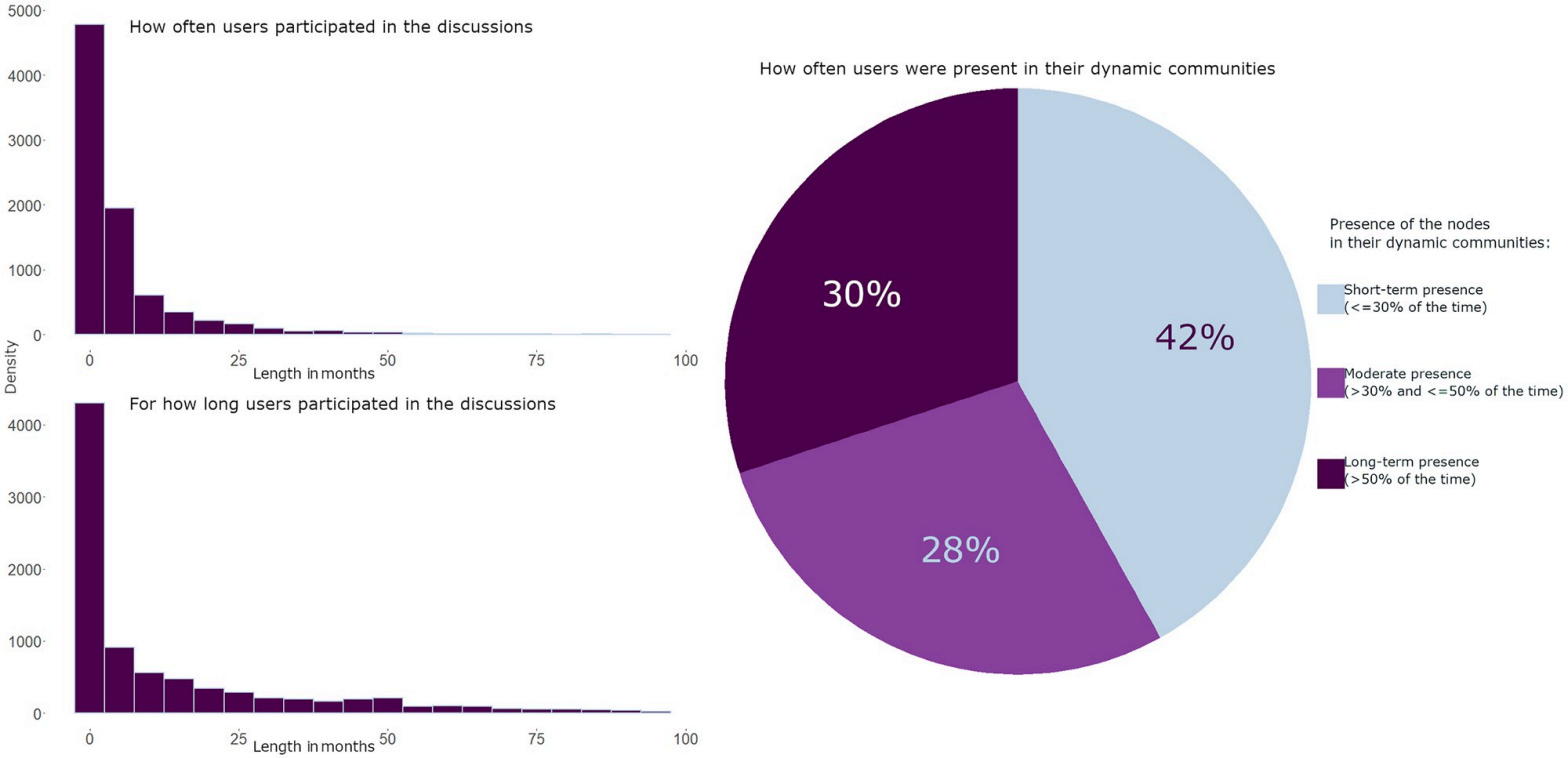
Statistics on the frequency (upper-left corner) and length (bottom-left corner) of users’ participation in the discussions, and the frequency of users’ community change (right side). *Notes*: The upper-left histogram shows the density of the users (on the Y-axis) who participated in online discussions for the number of months (*N*_*months*, *when the user was active*_) specified on the X-axis. The bottom-left histogram shows the density of the users (on the Y-axis) who participated in online discussions over the period (*T*_*last*_ − *T*_*first*_, where *T*_*last*_ is the last and *T*_*first*_ is the first months of user’s activity) of the number of months specified on the X-axis. The pie-chart of the right-hand side shows the proportion of users (i.e., the area of the pie portion on the figure), who stayed in their dynamic communities the proportion of months ($y=\frac{N_{months,\;which\;user\;spent\;in\;its\;dynamic\;community}}{\sum_{i=1}^{n}N_{months,\;which\;user\;spent\;in\;community\;i}}$, where *n* is the number of communities, in which the user was active over the examined period of time) specified by the colour of the pie portion. *N of users* = 8451 users. The communities were distinguished using iterative detection and matching [98].

**Fig 6:**
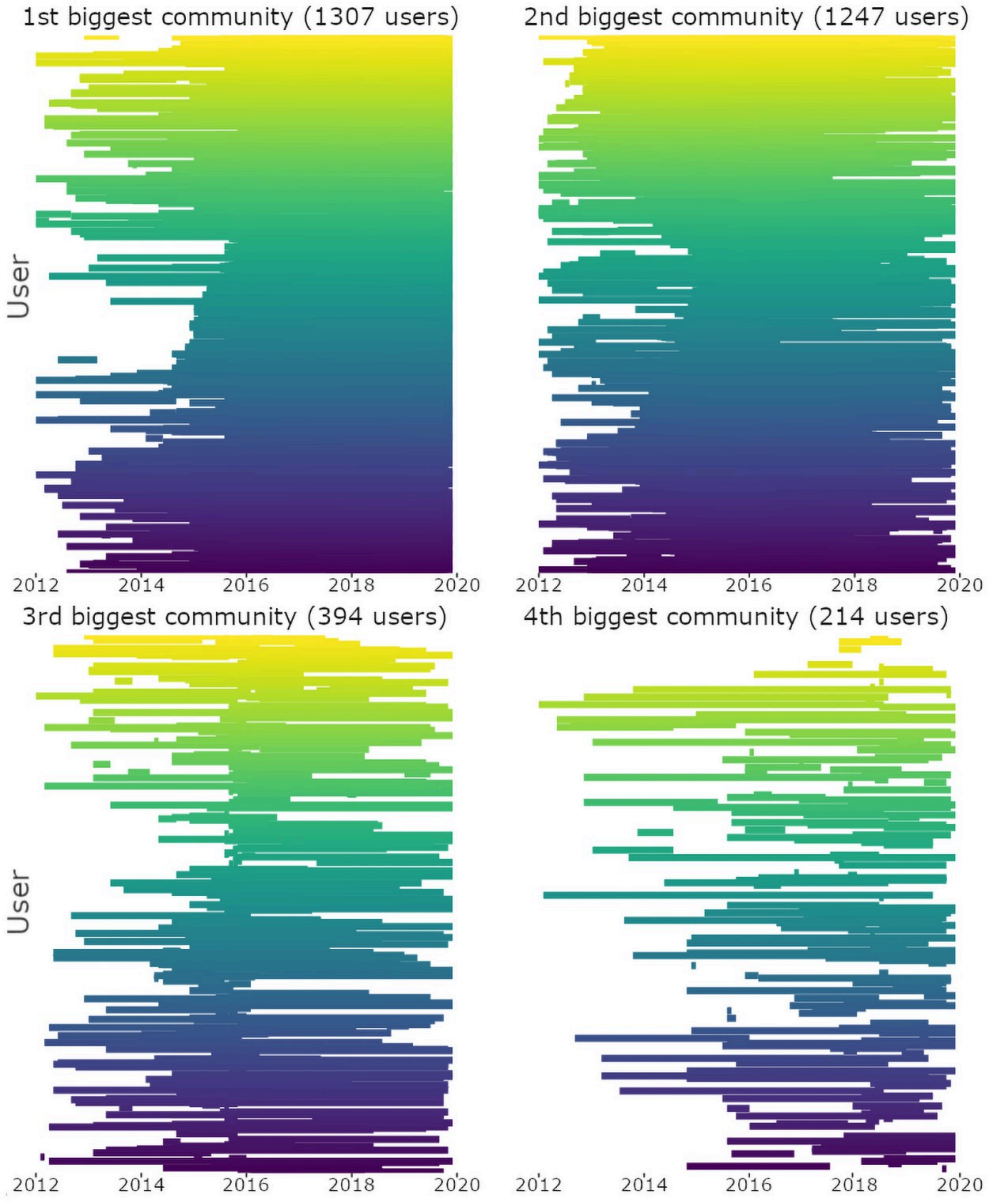
Users’ participation in Twitter discussions in the 4 biggest dynamic communities. *Notes*: Y-axis shows the users’ activity over the examined period (X-axis). Each row represents the activity of one user. *N of users* = 3162 users.

Moreover, Fig 5 suggests often infrequent and short-term participation of the users in their dynamic communities. Additionally, many users (42%) were joining different dynamic communities each month and seemed to interact with the members of their dynamic communities less than 30% of the time. The issue of infrequent participation in political discussions on social media platforms was widely addressed previously [39, 40]. Fig 5, in part, may support the reference to online social activism as to “slacktivism”. On the other hand, Fig 5 also shows that many users (30%) stay in their dynamic communities more than 50% of the time, participate regularly (see the upper-left histogram of Fig 5) and for a long period of time (more than 2 years on the bottom-left histogram of Fig 5). In particular, users of the biggest communities seem to exhibit long-term participation and frequent engagement with other participants (see Fig 6).

**Fig 7:**
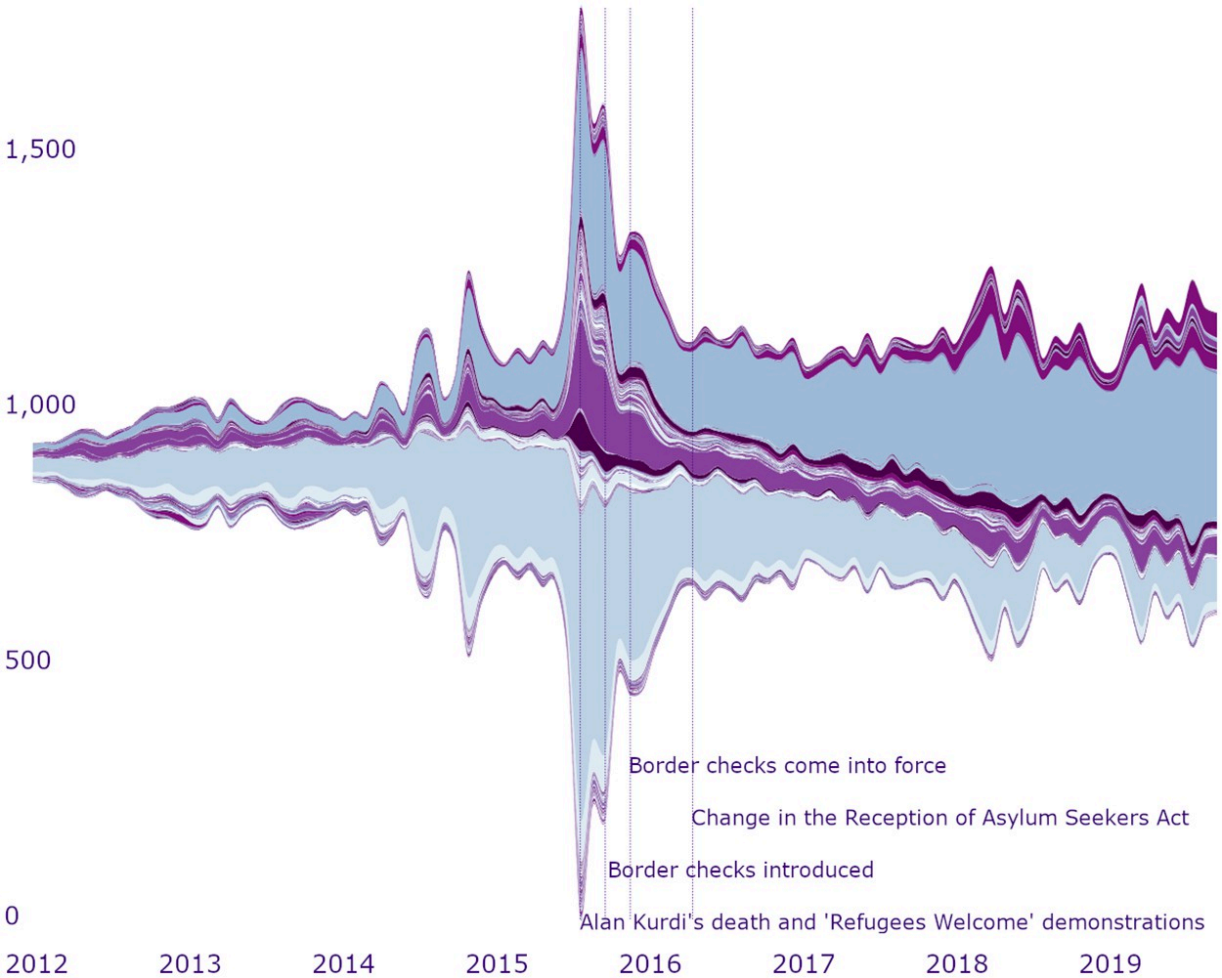
Growth of user communities in time. *Notes*: The entities show the dynamics of the community size change. Y axis shows the number of users in dynamic communities at each of the time point. *N of time points* = 96 (months). *N of communities* = 722 communities. *N of users* = 5602 users. The streamgraph visualisation technique was used to present the growing number of users in the dynamic communities [108]. A streamgraph is a type of stacked area graphs, where values are plotted around a varying central baseline [109]. Such a visualisation technique allows examining dynamic changes in the data. The colour palette is used to differentiate between the dynamic (i.e., temporal) communities. The communities were distinguished using iterative detection and matching [98].

Within the examined period, ten biggest communities emerged in January 2012, however, they reached the maximum size in the second half of 2015 and expanded at the end of 2017. This may be explained by the steady inflow of content-creators (see Fig 7) to the network, which can be associated with the growing popularity of social media in general, and Twitter in particular. Nonetheless, it is evident that the communities’ rapid expansion coincides with the major political events in the country, such as national elections in the fall of 2014 and 2018, the news about the tragic death of Alan Kurdi, or the introduction of border controls on the Swedish border with Denmark.

**Fig 8:**
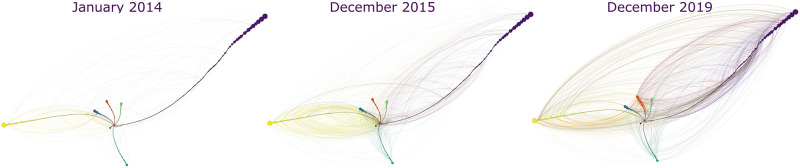
Interactions between the users of the 10 biggest communities over time. *Notes*: The entities show the users of the 10 biggest dynamic communities as nodes and connections (i.e., replies and mentions) between those users as arcs at three time points. *N of users* = 989 users. Groeninger’s radial axis layout within the Gephi environment [110] was used to visualise the network. Here, the nodes are grouped according to their dynamic communities, which are presented as whiskers. Each whisker (i.e., community and the nodes within this community) has its own colour to differentiate between the dynamic communities. The arcs have the colour of the users mentioning or replying to a user from another community.

Examining the interactions (i.e., arcs) between the users belonging to different dynamic communities (see Fig 8), we can see that the users from the ten biggest communities tend to frequently communicate with the members of other communities. Moreover, the number of interactions substantially increases in the midst of the crisis and stays high at the end of the examined period. This can be explained by the inflow of new users in 2015 (see Fig 1). Still, Fig 8 shows that the biggest dynamic communities are hardly echo-chamber-like groups of users. In general, the data shows that only 24% of all communities with three or more users can be considered as pure echo-chamber environments.

**Fig 9:**
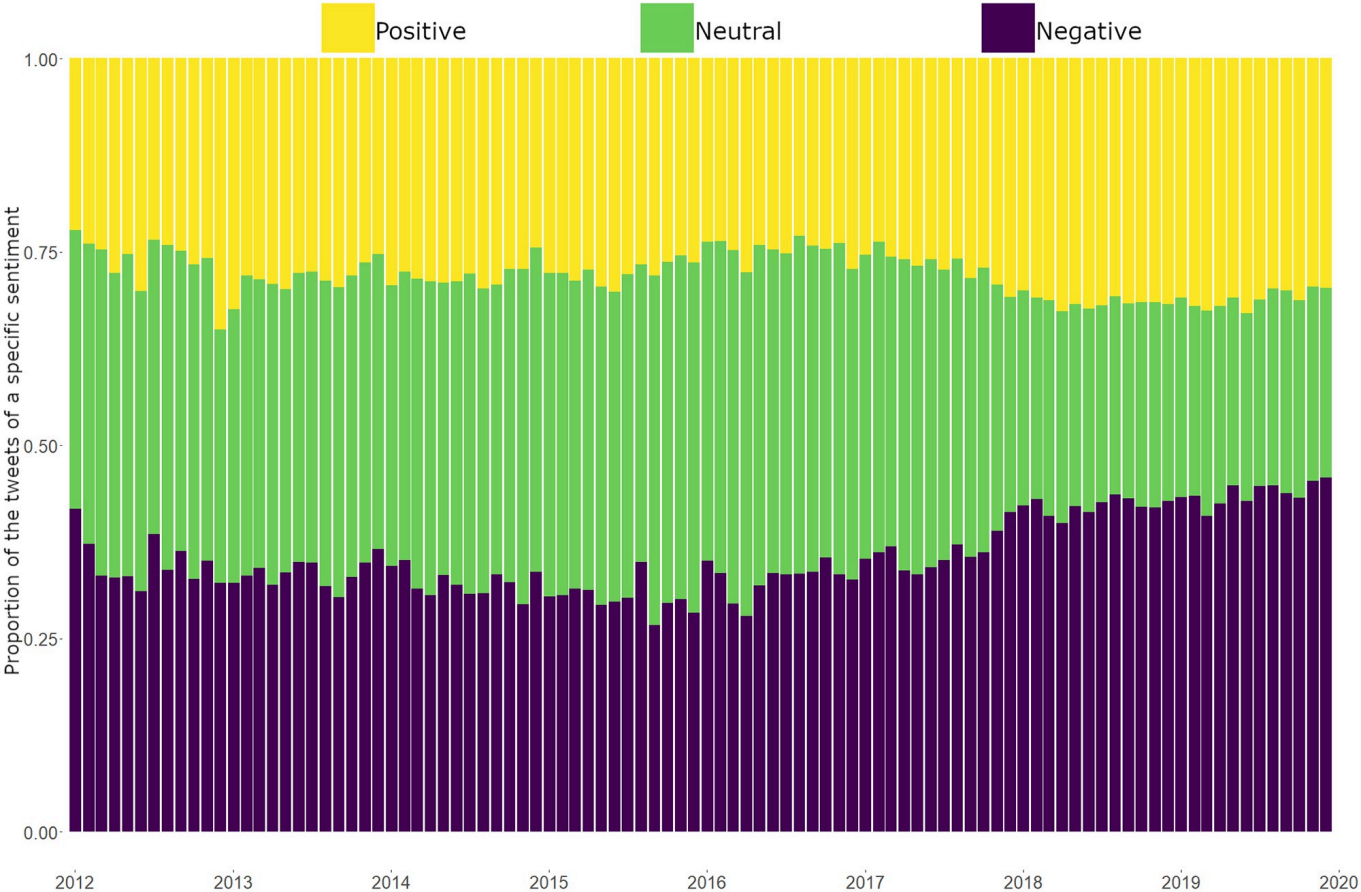
Ratio of negative and positive tweets. *Notes*: The bars show the ratio of the tweets with negative, positive or neutral sentiment. *N of tweets* = 686 763.

**Fig 10:**
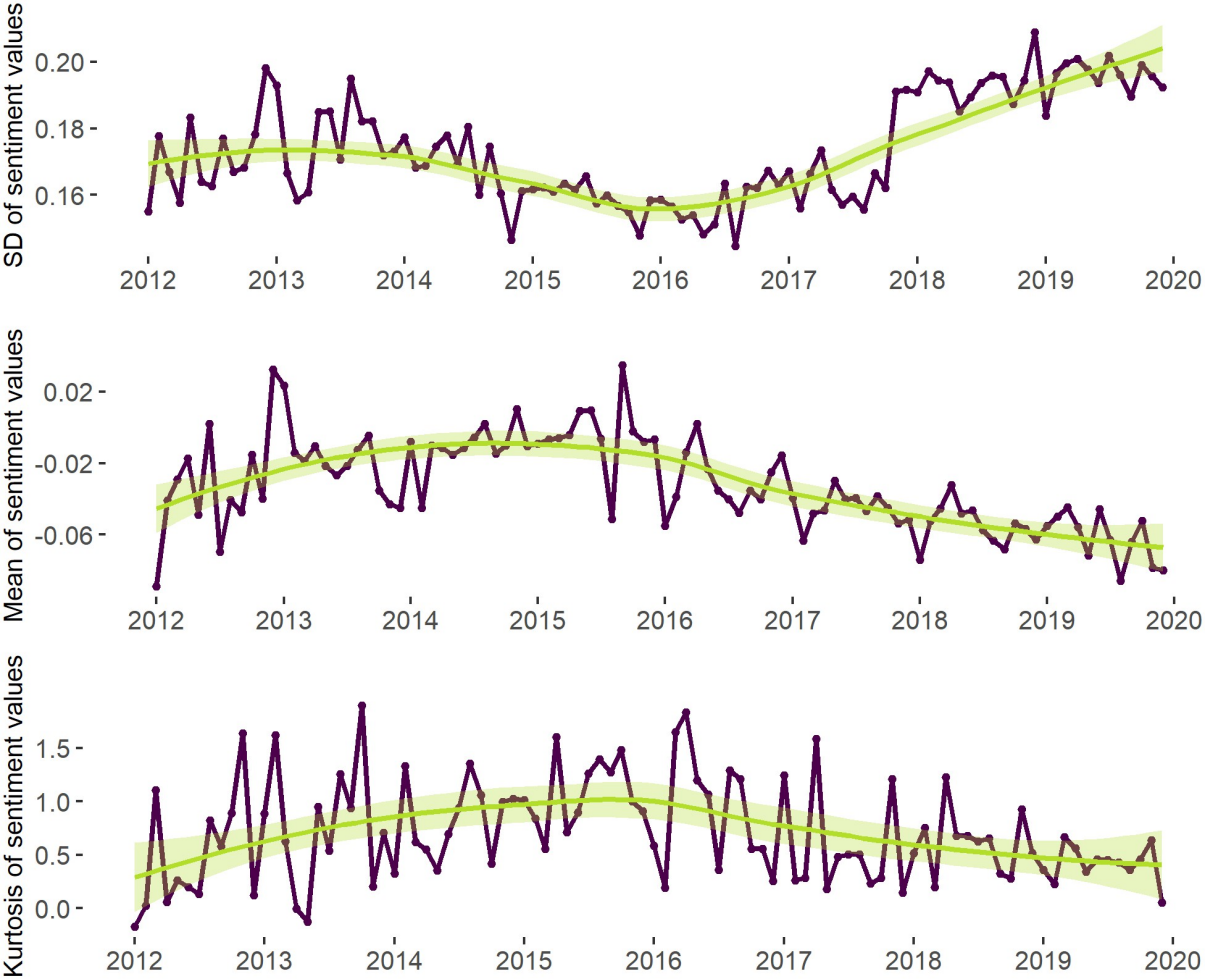
Mean, standard deviation and kurtosis of the sentiment values. *Notes*: The mean, standard deviation and kurtosis are calculated as follows: $\bar{X_{i}}\;=\;\frac{\sum_{ki=1}^{N_{i}}X_{ki}}{N_{i}}$; $\sigma {}_{i}\;=\;\sqrt{\frac{\sum_{ki=1}^{N_{i}}(X_{ki}-\bar{X_{i}}{)}^{2}}{N_{i}-1}}$; $\gamma {}_{2i}\;=\;\frac{\sum_{ki=1}^{N_{i}}(X_{ki}-\bar{X_{i}}{)}^{4}}{N_{i}\sigma^{4}}-3$ where $\bar{X_{i}}$ is the mean of the sentiment values at month *i*, *σ*_*i*_ is the standard deviation of the sentiment values and *γ*_2*i*_ is the kurtosis of the sentiment values, *N*_*i*_ is the number of users at month i and *X*_*ki*_ is the sentiment of *k* user. The regression line (within the *ggplot2* package [111]) is fitted into the points measuring the mean, standard deviation and the kurtosis of the sentiment values within each time point. *N of time points* = 96 (months). *N of tweets* = 686 763. *N of users* = 9 300. Valence Aware Dictionary and sEntiment Reasoner (VADER) [103] was applied to the tweet texts to extract sentiment values.

**Fig 11:**
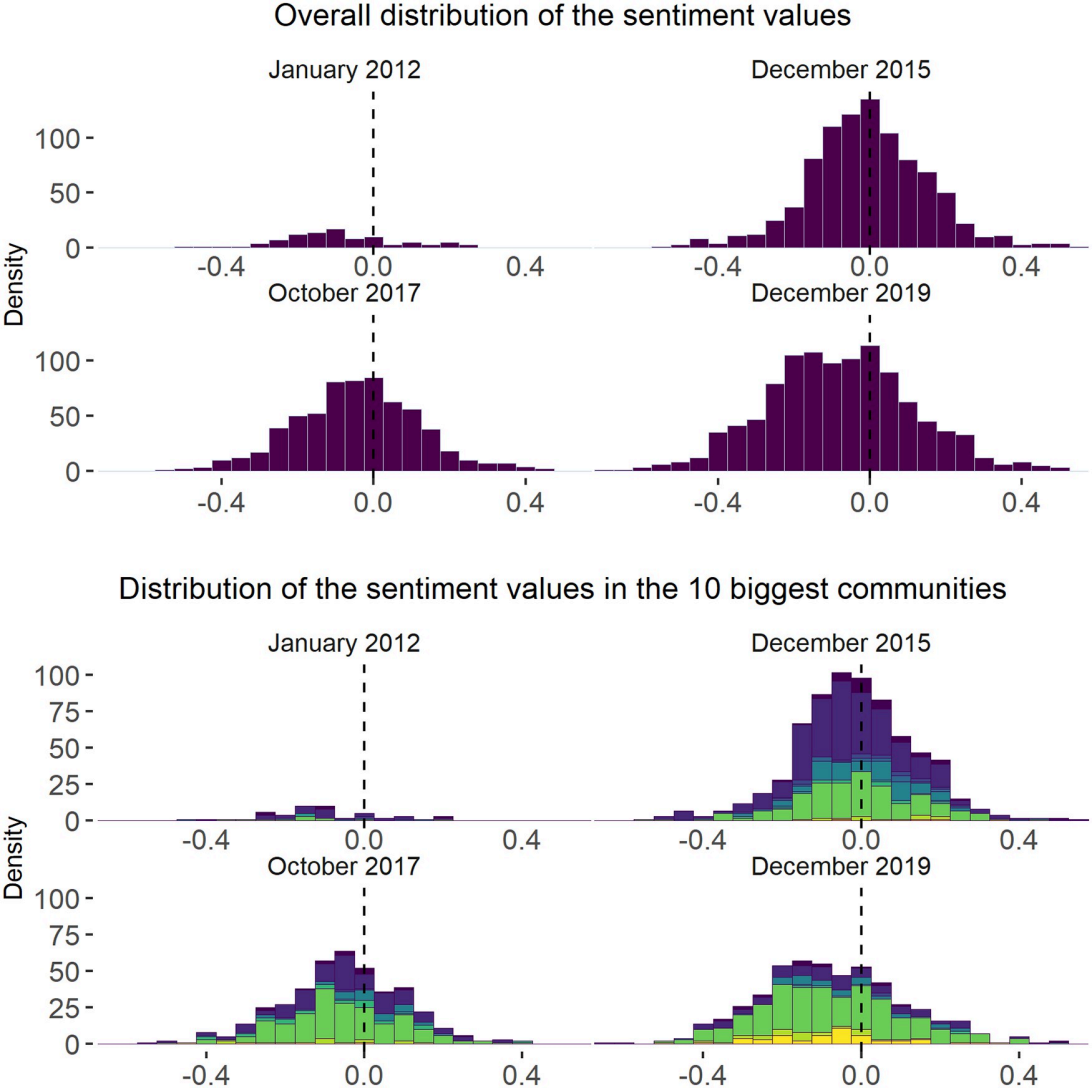
Density of the sentiment values at 4 time points. *Notes*: Density plots were built using the tools of the *ggplot2* package [111]. Stacked density plots demonstrate distribution of the sentiment values within 10 biggest dynamic communities (four figures at the bottom) and in the whole network (four figures at the top). The figures show the distribution of the sentiment values calculated per user in a given month (i.e., $X_{ki}=\frac{\sum_{lki=1}^{n_{ki}}x_{lki}}{n_{ki}}$, where *X*_*ki*_ is the sentiment of user *k* at month *i*, *n*_*ki*_ is the number of tweets written by that user and *x*_*lki*_ is the sentiment of each *l* tweet written by that user). *N of time slices* = 96 (months). *N of dynamic communities* = 10. *N of tweets in the network* = 686 763. *N of tweets in the communities* = 491 891.

Analysing the ratio of negative and positive tweets within the study period (see Fig 9), we can see a declining share of neutral tweets and a steadily growing share of negative messages after the second half of 2016, which suggests users’ growing inclination to post negatively toned tweets. This observation is also supported by the decreasing mean of sentiment values (see Fig 10) and the noticeable changes in the distribution of sentiment values at the end of 2017 (see Fig 11). Thus, we can see that 2017 can be characterised by a shift towards more negative sentiments in our discussion network.

Moving further to the study of polarisation dynamics in our network, previous research has suggested that bi-modality of the distribution of opinions or sentiments can be described as one of the dimensions of polarisation [112]. To obtain evidence of polarisation in our network, we conducted Hartigan’s Dip test of unimodality of the sentiment values’ distributions for each month [106]. The results of Hartigan’s Dip test for all 96 time-slices allow contending that the distributions of sentiments on the network level are unimodal (p-value > 0.05), which gives evidence against the presence of polarisation on the network level.

Hartigan’s Dip test was further supplemented by the visual inspection of the changes in the sentiments’ distributions on the network and community levels. Indeed, Fig 11) also demonstrates no division of the sentiment values into two groups (groups of positive and negative tweets), which, once again, serves as an evidence against the presence of bi-modality in the data. In fact, December of 2015 is associated with the growth of kurtosis and peakier distribution when compared to the other 3 time points (2012, 2017 and 2019). Moreover, Fig 10 shows kurtosis of the sentiment distribution reaching maximum values in 2015, which also suggests no short-term polarising effect of the 2015 events. While these results do not support the suggestion about the growth of polarisation on the network level as a result of the refugee crisis, we find no evidence to accept (H1) or (H2).

As suggested above, the dispersion or spread of sentiment values can be described as one more dimension of polarisation. Fig 10 demonstrates the changes in the standard deviation (SD) of sentiment values in 2012–2019. Indeed, while the spread of sentiments slightly decreases in 2015, in the midst of the crisis, it starts to slightly increase in 2016, but undergoes a major rise only in the second half of 2017. Moreover, in 2015, the general sentiment was closer to the centre of the distribution and to 0 (as Fig 11 shows), suggesting that the expression of less extreme sentiments. In the meanwhile, after 2016, the kurtosis values steadily fall, which, together with the above-mentioned observation about the flatter shape of sentiment distribution, gives some evidence for growing sentiment diversity in the network.

**Fig 12:**
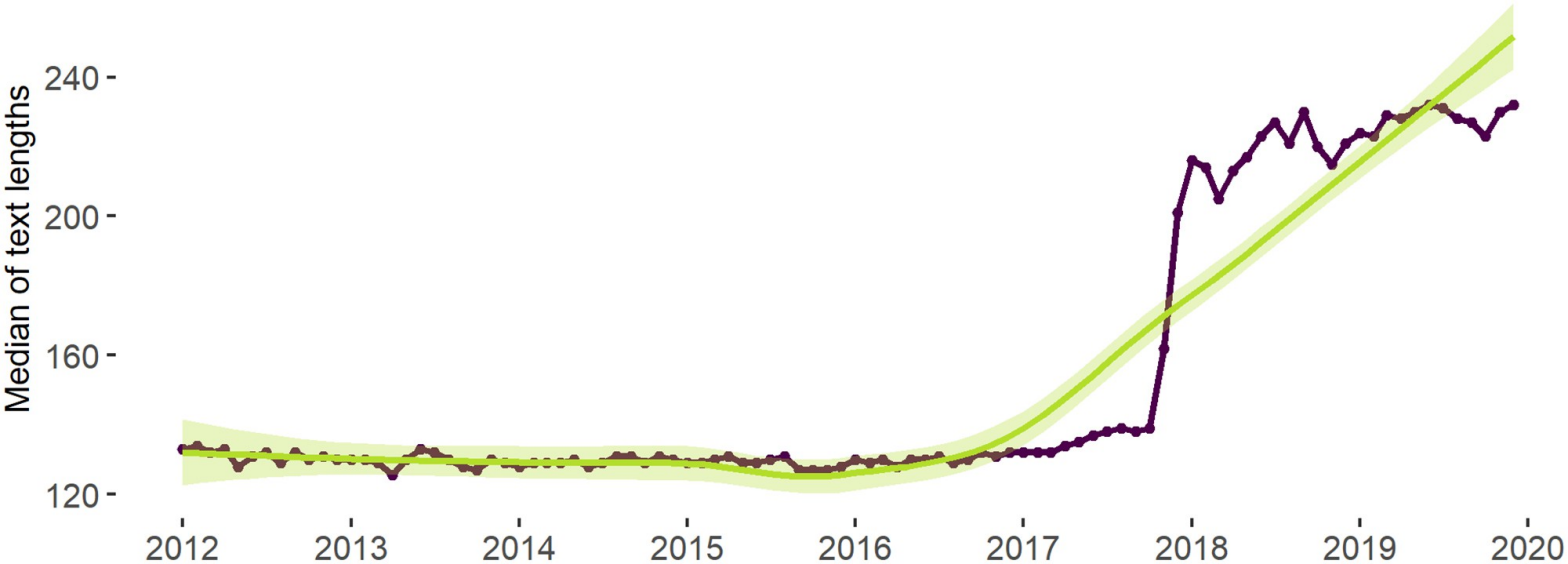
Median of the tweet length over time. *Notes*: The line is fitted into the points showing the values of the tweet length median in a given month. The length of a tweet post is the number of characters in the tweet text. *N of tweets* = 686 763. *N of time slices* = 96 (months).

**Fig 13:**
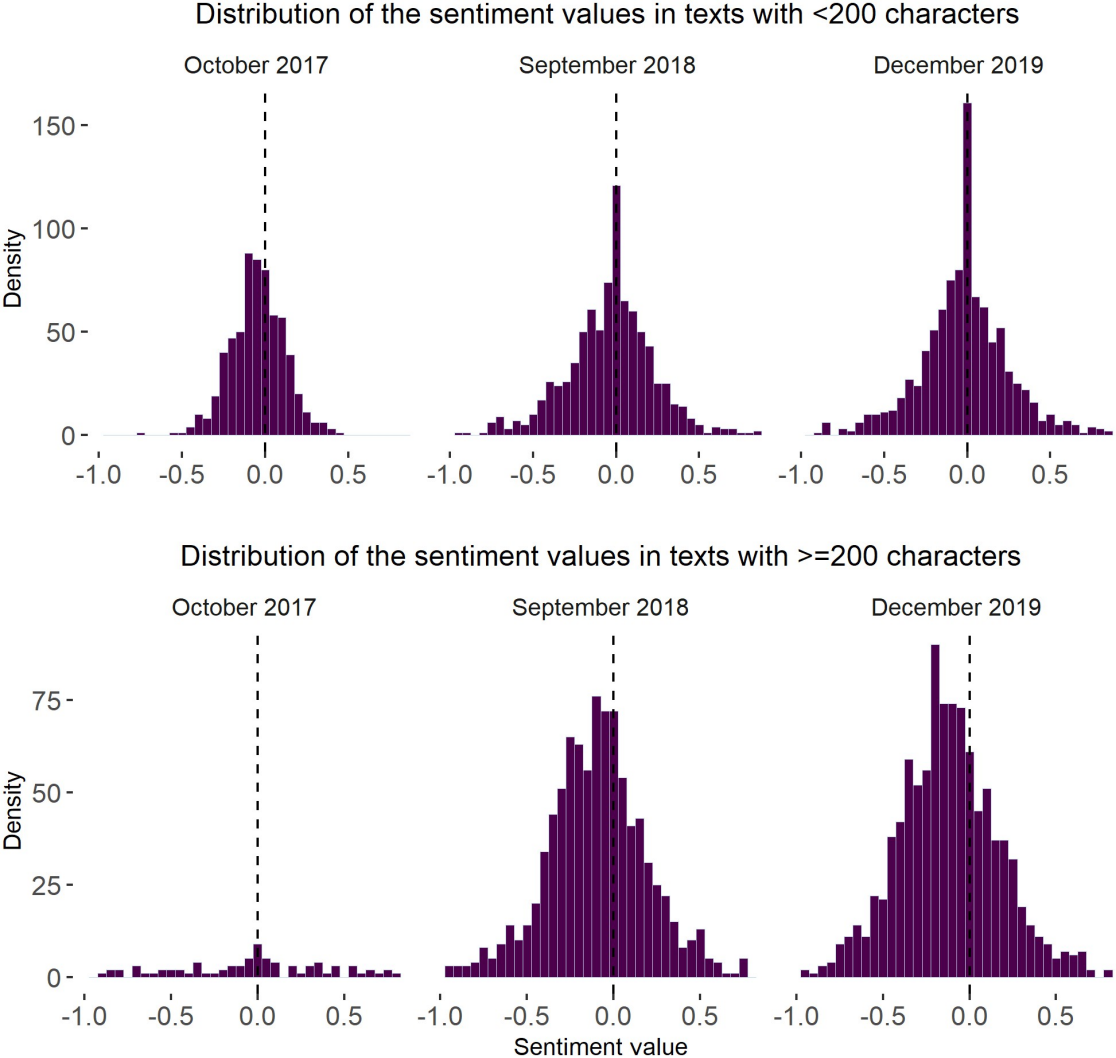
Distribution of sentiment values in the tweets of less and more than 200 characters. *Notes*: Density plots were built using the tools from the *ggplot2* package [111]. Three figures at the top show the distribution of sentiment values in the tweets with less than 200 characters. Three figures at the bottom show the distribution of sentiment values in the tweets with > = 200 characters. *N of time slices* = 96 (months). *N of tweets* = 686 763.

These results may suggest that the years after the refugee crisis are associated with the long-term changes in the sentiment dynamics, and in particular, with the increased spread of user sentiments as one of the dimensions of polarisation. It is not clear, however, if those sentiment changes can be characterised as significant ones or whether they are directly linked to the refugee crisis. In fact, one of the possible explanations could be the revision of Twitter’s policy on the maximum tweet length. In particular, at the end of 2017, the company increased the limit on tweet characters from 140 to 280 [113]), which could have caused subsequent adjustments of the patterns in the way people express themselves. Indeed, Fig 12 shows that, on average, when given the opportunity to write longer tweets, people used this opportunity and tweeted messages of more than 200 characters. Comparing the distributions of the sentiment values in tweets of less and more than 200 characters, one can find that the sentiment distributions in longer tweets tend to have heavier tails and flatter shape (see Fig 13). This result may suggest that text length influences how people express themselves.

Indeed, previously, Twitter’s representatives admitted that users became more engaged with the platform after the changes in the tweet length policy: “In addition to more Tweeting, people who had more room to Tweet received more engagement (Likes, Retweets, @mentions), got more followers, and spent more time on Twitter” [114]. Earlier, Gligorić, Anderson and West [115] also reported semantic and topic differences between longer and shorter tweets. Examining Fig 13, one can also identify a positively skewed distribution of sentiments in longer tweets, which suggests that tweets of more than 200 characters often have more negative sentiment in comparison with the short one. This observation suggests that internal modifications in the social media platform’s architecture could have led to changes in users’ behaviour and the discussion dynamic.

**Fig 14:**
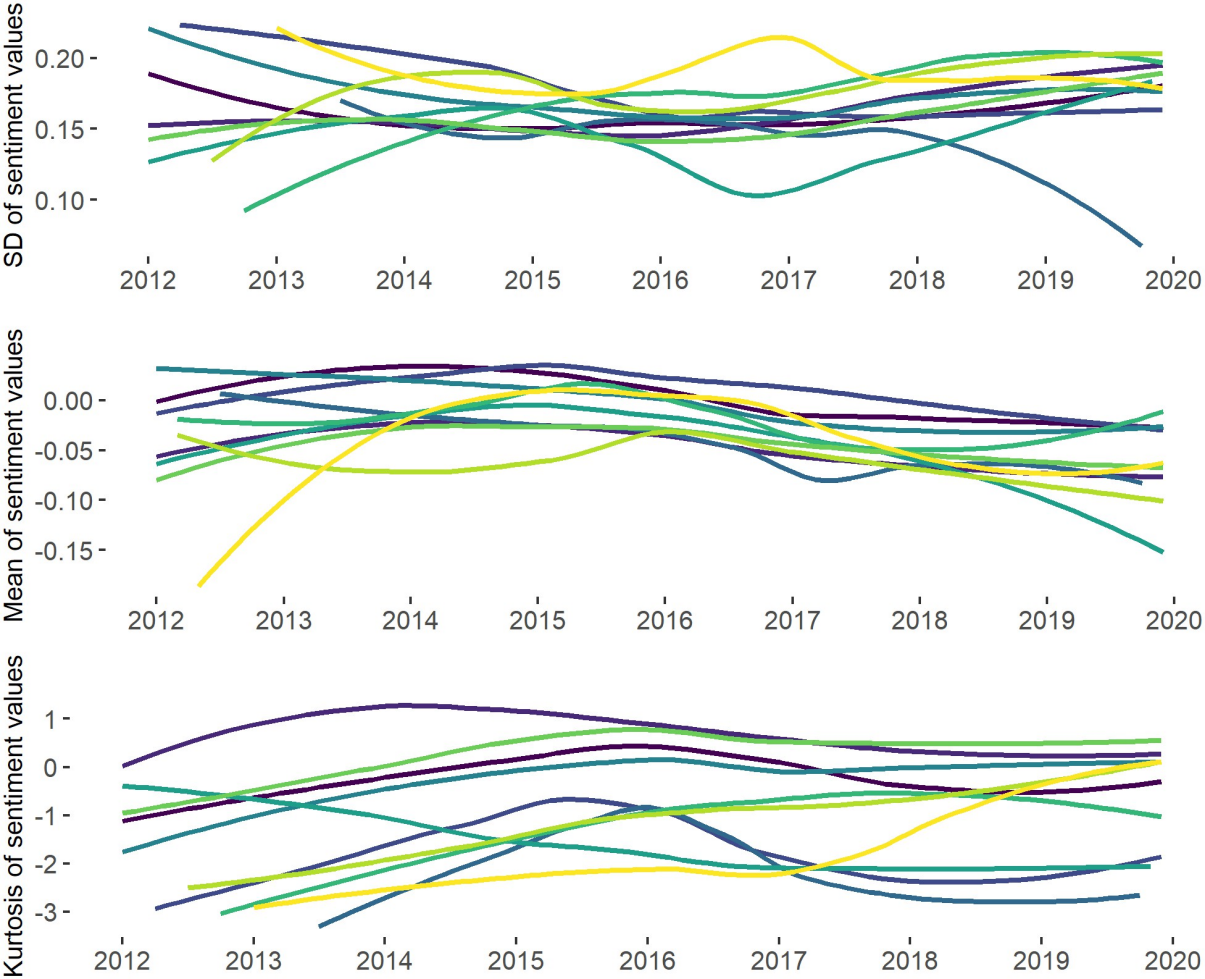
Mean, standard deviation and kurtosis of sentiment values in the biggest dynamic communities. *Notes*: The regression line (within the *ggplot2* package [111]) is fitted into the points measuring the mean, standard deviation and kurtosis of the sentiment values within each dynamic community in the examined time-period. *N of time slices* = 96 (months). *N of dynamic communities* = 10. *N of tweets* = 491 891.

The study of sentiment dynamics on the community level demonstrates patterns similar to those on the network level. In particular, Fig 14 shows similar patterns discussed in regard to the overall sentiment distribution visible in all big communities. Thus, once again, one can find the spread of the negative sentiment in all communities after 2016, the decrease of the kurtosis values at the same period and the growth of the standard deviation in 2017. Moreover, Fig 11 shows that the distributions of sentiment values in the biggest communities in the four key periods resemble those on the network level. The results of Hartigan’s Dip test also suggest no polarisation on the community level throughout the study period. Thus, on the community level, we also find no evidence to accept (H1) and (H2).

**Fig 15:**
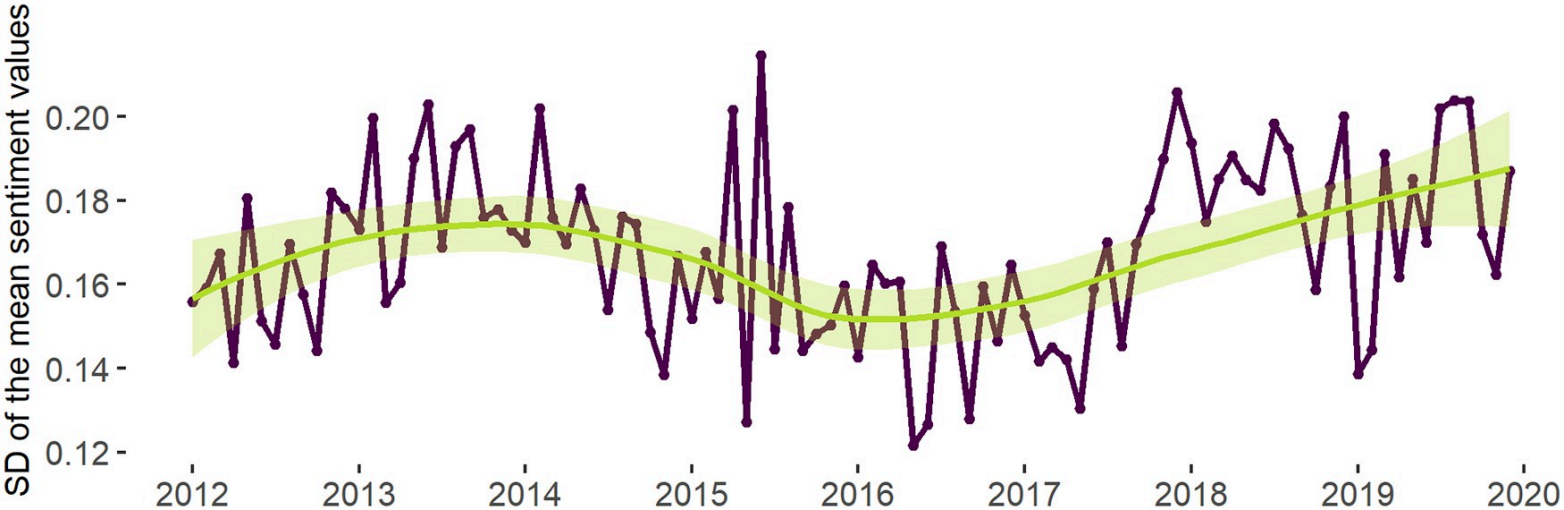
Standard deviation of mean sentiment values in the dynamic communities. *Notes*: The line (within the *ggplot2* package [111]) is fitted into the points measuring the standard deviation of the mean sentiment values in all of the dynamic communities. *N of time slices* = 96 (months). *N of dynamic communities* = 723. *N of tweets* = 565 888.

Fig 15, however, once again, shows 2016 to be associated with the lowest standard deviation of sentiment values in all dynamic communities if compared to other time-points. After 2016 the standard deviation gradually increases and reaches maximum by 2020. That suggests the increasing spread of the sentiment values between dynamic communities (i.e., the same spread of the distribution that we found at the network level).

**Fig 16:**
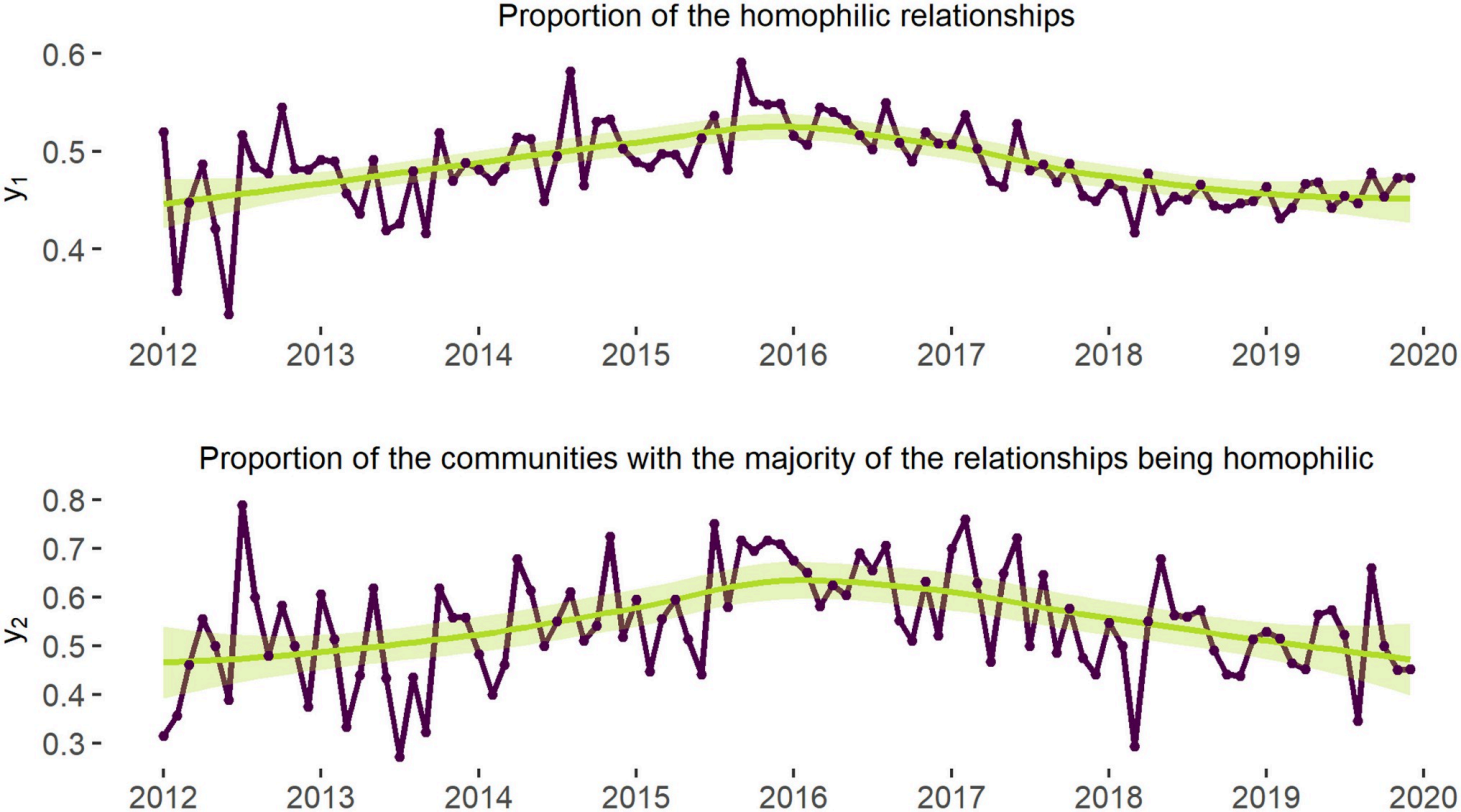
Ratio of homogeneous edges in the network and ratio of network’s communities with the majority of homophilic relationships. *Notes*: The line is fitted into the points measuring the specified proportion for each month. $y_{1}\;=\;\frac{N_{connections\;between\;the\;users\;with\;similar\;views}}{N_{total\;number\;of\;the\;connections}}$. $y_{2}\;=\;\frac{N_{communities\;with\;the\;majority\;of\;homophilic\;edges}}{N_{total\;number\;of\;communities}}$. Higher values identify the growing number of connections between the users expressing similar views in the network as a whole and in its dynamic communities. Only those communities that consist of more than 3 users were considered to visualise the the proportion of the network’s communities with the majority of the relationships being homophilic. *N of time-periods* = 96 (months). *Total N of edges* = 682 821. *N of communities* = 70.

To measure the level of sentiment homophily among the users (in relation to the third hypothesis (H3)), we calculated the ratio of connections between the users with homogeneous (e.g. positive—positive) and heterogeneous sentiments (e.g. positive—negative) (see Fig 16). In general, we see that, on the network level, every second edge is formed between other-minded users (see the “Proportion of the homophilic relationships” on Fig 16), which supports our earlier observation that this discussion network is far from being an echo-chamber and allows exposure to alternative sentiments among the users. On the community level, though, we can find more than half of all communities to be characterised by the prevailing sentiment homophily (see the “Proportion of the communities with the majority of the relationships being homophilic” on Fig 16). These results give evidence in favour of (H3) that predicts sentiment homophily on the community level.

In the meanwhile, at both the network and community levels, we can find the number of homophilic relationships to decrease after 2016. In fact, the shapes of the lines fitted into points showing the number of homophilic relationships (i.e., $y_{1}\;=\;\frac{N_{connections\;between\;the\;users\;with\;similar\;views}}{N_{total\;number\;of\;the\;connections}}$) and the number of homophilic communities per month ($y_{2}\;=\;\frac{N_{communities\;with\;the\;majority\;of\;edges\;being\;homophilic}}{N_{total\;number\;of\;the\;communities}}$) repeat the line of the kurtosis value changes at the network (Fig 10) and community (Fig 14) levels. This suggests that, while the number of people expressing more negative sentiments grows (which is also supported by the statistics depicted by Fig 15), the users become more likely to interact with other-minded people.

## Discussion

Summing up the findings outlined in the previous section, we saw that the vast majority of users remained rather passive in the discussions on the topic—out of more than the initial eighty-five thousand users present in the data set, less than ten thousand participated in the discussions with others on a frequent basis. This observation is in line with the results of previous research that has described the majority of social media users as content-consumers rather than agents capable of driving discourse changes on the social media platforms [41]. Moreover, more than one-third of active users were not assigned to any of the dynamic communities, which means that their contact with other users was mostly segmentary and infrequent. Despite the fact that immigration can be seen as a controversial issue lacking consensus (especially in the European context), we found no pronounced polarisation neither on the network nor on the community levels. As outlined in the introductory section of the paper, we expected to detect the growth of users’ polarisation directly after the peak of the crisis (H1), as suggested by the previous research [32, 33]. The first hypothesis was underpinned by the idea that the events associated with the crisis (e.g., the tragic death of Alan Kurdi or sexual assaults in German Cologne at the end of 2015) would trigger diversified responses and reactions from the social media users as well as further problematise Sweden’s existing immigration and integration policies. Our hypothesis was not confirmed. Thus, we have not found any major changes in the levels of sentiment dispersion or modality of the sentiments’ distributions neither on the network nor on the community level directly after the peak of the crisis at the end of 2015.

Even more so, we have seen that the dispersion of sentiment values has become smaller in the wake of the crisis. That suggests that the users tended to articulate more unanimous and less extreme sentiments on the immigration topic, which could actually speak in favour of depolarising effect of the crisis on the users’ sentiment. Relating these observations back to the theoretical assumptions outlined in the introductory section of the paper, we suggest that outstanding events and crises do not necessarily cause growing polarisation of users’ sentiments, which to some extent contradicts the existing (although quite limited) evidence [32, 33].

Our second hypothesis (H2) was based on the argument that the refugee crisis can be seen as a watershed moment that made previously marginalised racist and populist narratives and frames of the immigration issue normalised in the public debate [see, e.g., 116] and that the crisis brought about persistent changes in users’ sentiment polarities as suggested by [31]. These expectations, however, were mostly not confirmed—we detected no permanent changes in the levels of polarisation that could be directly attributed to the crisis, which applies both to the network and community levels. Still, we saw a moderate but long-lasting shift towards a more negative tonality of users’ messages after the crisis and a declining share of neutral tweets, which can be seen as one of the negative effects of the crisis on the sentiment dynamics and which may suggest that users became more sceptical with regard to the immigration topic. However, we have also observed that the growth of sentiment diversity in the end of 2017 coincides with Twitter’s decision to extend tweet length. One may suggest that the latter gave the users more space to articulate their sentiment (since it may, indeed, be problematic to express a sentiment or subjective judgment on the discussion topic in just 140 characters). Thus, the growing sentiment diversity can also be a product of the changes in the platform architecture rather than an independent shift in the sentiment dynamics.

As argued in the introductory section of this paper, we focused our analysis on two different dimensions of polarisation, namely, dispersion (opinions’ diversity) and bi-modality (opinions’ distinctness). As of the latter, we saw that users’ sentiments cannot be characterized as distinct since there exists no gap in the users’ sentiments with regard to the immigration topic. As for opinion (sentiment) diversity, we have seen a certain move towards the negative and more extreme end of the polarity spectrum. However, as mentioned above, it is not clear whether this development may depend on the changes in tweet length. Nevertheless, as predicted by the literature on the attitude and opinion polarisation and given decreasing average sentiment, the growing opinion or sentiment repertoire can be seen as a condition that prevents the political system’s ability to reach consensus [82, p.693]. In the years to come, it will be interesting to see whether this would play out in the case of Swedish immigration policies and political parties’ ability to agree upon them.

Our third hypothesis was grounded in the vast literature on selective exposure and homophily in online networks [among others, 7]. In our empirical case, we have seen that sentiment homophily can be perceived as a factor for community building, which also allows us to confirm the third hypothesis (H3). In particular, we saw that homophilic relations among the users were more common than heterophilic ones, while the absence of strong polarisation patterns within the communities also gives evidence in favour of (H3). Thus, although we find no evidence for a pure echo-chamber-like effect of interactions in the communities, the mechanism of selective exposure seems to be more dominant on the community level, which supports the earlier findings [7]. On the other hand, the users still interact with those outside of their own communities, which compensates homophily on the community level and allows users’ exposure to alternative sentiments.

Finally, we would like to finish this section by mentioning some of the limitations of our study. First of all, this article builds upon an unsupervised method for sentiment analysis represented by the VADER tool. While it provides better quality of final classifications in comparison with standard lexicon-based methods, it is nevertheless less accurate than supervised approaches, especially given that the Swedish version of VADER uses machine translation of the words in the dictionary. Furthermore, another concern is that documents with no terms included it the lexicon automatically receive a score of zero, which makes it problematic to distinguish between documents with “real” neutral sentiment and those where no key-words could be identified. On the other hand, given the absence of labelled training data and short length of tweets, one can suggest that VADER definitely has its place among the text analytic methods.

The second limitation of our study comes from the fact that inactive users (i.e., content-consumers), as well as retweeted messages, were excluded from the analysis. That makes us unable to generalise the findings to the whole discussion network. In this analysis, we decided to focus on active users due to their potential ability to engage new users into a political discussion, radicalise masses [6, 45] and mobilise people into political action [44]. We chose 5 tweets per month as an arbitrary boundary between active and non-active users due to the fact that, to our knowledge, there is no accepted measurement of active participation in online discussions. This research design decision may have potentially affected the results of the study. Nevertheless, our decision to concentrate only on active users has also been dictated by the need to minimise the effects of the so-called “Ship of Theseus” in temporal networks that are generally characterised by high variability and instability [95].

Finally, the fact that Twitter revised the policy on the maximum tweet length in 2017, may have had an effect on the results of the study. Indeed, we found the distributions of the sentiment values in messages < 200 characters and > = 200 characters to have different shapes. In particular, the prevalence of negative sentiments is more visible in longer tweets. Thus, we suggest that the internal modifications in the social media platform’s architecture may have become an obstacle in tracking the long-term polarisation changes within the study period and finding evidence to support (H2).

## Conclusion

In this paper, we contributed to a theoretical discussion on the mechanisms and dynamics of polarisation in social-media-driven networks. We focused on the role of external crises as potentially polarising events able to serve as watershed moments for users’ sentiments and on the immigration topic as a socially relevant and potentially polarising issue. We conclude that, in general, the European refugee crisis had only a limited and short-term effect on the polarisation dynamics, while, on the other hand, it seems to have had negative effect on the general tonality of users’ messages. The next steps in the research on this topic could be to study the polarisation dynamics on the different steps of dynamic communities’ life cycles and the effects of users’ engagement and frequency of participation on the dynamics of discussions. Moreover, it would be interesting to examine such dynamics in the communities formed in the social media platforms other than Twitter, to avoid the pronounced effect of the platform architecture on the results of the study. This would provide a better understanding of the role of social media platforms in the reduction of radicalisation and right-wing populism.

## Supporting information

S1 Fig The step-wise analysis of the retrieved data. (TIF)

S2 Fig The results of the dynamic partition evaluation. The Fig on the top left represents modularity at each step (X-axis) if one of the algorithms (Y-axis) is applied. The Fig on the top right represents consecutive similarity (X-axis) if one of the algorithms (Y-axis) is applied. The Fig on the bottom is the comparison of the algorithms depending on the global smoothness scores (Y-axis): from left to right, those are the average value of partition smoothness, node smoothness and label smoothness (X-axis). The figures evaluate the partition of the dynamic network into communities as the result of applying iterative detection and matching [98], label smoothing [99] and smoothed Louvain [100]. See the detailed description of the algorithms in [97].
(TIF)

S3 Fig Dynamic community birth, death and peak in terms of the number of users by month. Y-axis shows the number of communities that emerged, died and reached the peak in terms of the number of users. The communities are received after applying iterative community detection using Clauset-Newman-Moore greedy modularity maximization and matching [97].
(TIF)

S4 Fig User participation in Twitter discussions in 4 biggest dynamic communities. Y-axis shows the users activity over the examined time-period (X-axis). Each row represents the activity of one user.
(TIF)

S5 Fig Top 20 words associated with the tweets expressing positive and negative opinions about migration in Sweden. Term-category association (TCA) [105] analysis was applied to identify the words associated with the tweets expressing positive and negative opinions about migration in Sweden. *N of tweets* = 686 763.
(TIF)

S1 File Supplementary information. The document provides additional information on the data handling and methods used for the analysis and complements the main text of the manuscript.
(ZIP)

S2 File Supplementary R/Python script. The R/Python script used for the analysis can be found at this link: https://elizabethkopacheva.github.io/CET-W2V/.
(TXT)
