# Supplementary figures and images for: Users’ polarisation in dynamic discussion networks: The case of refugee crisis in Sweden
Source: PLoS One. 2022 Feb 9;17(2):e0262992. doi: 10.1371/journal.pone.0262992 (PMC8827437; doi:10.1371/journal.pone.0262992)

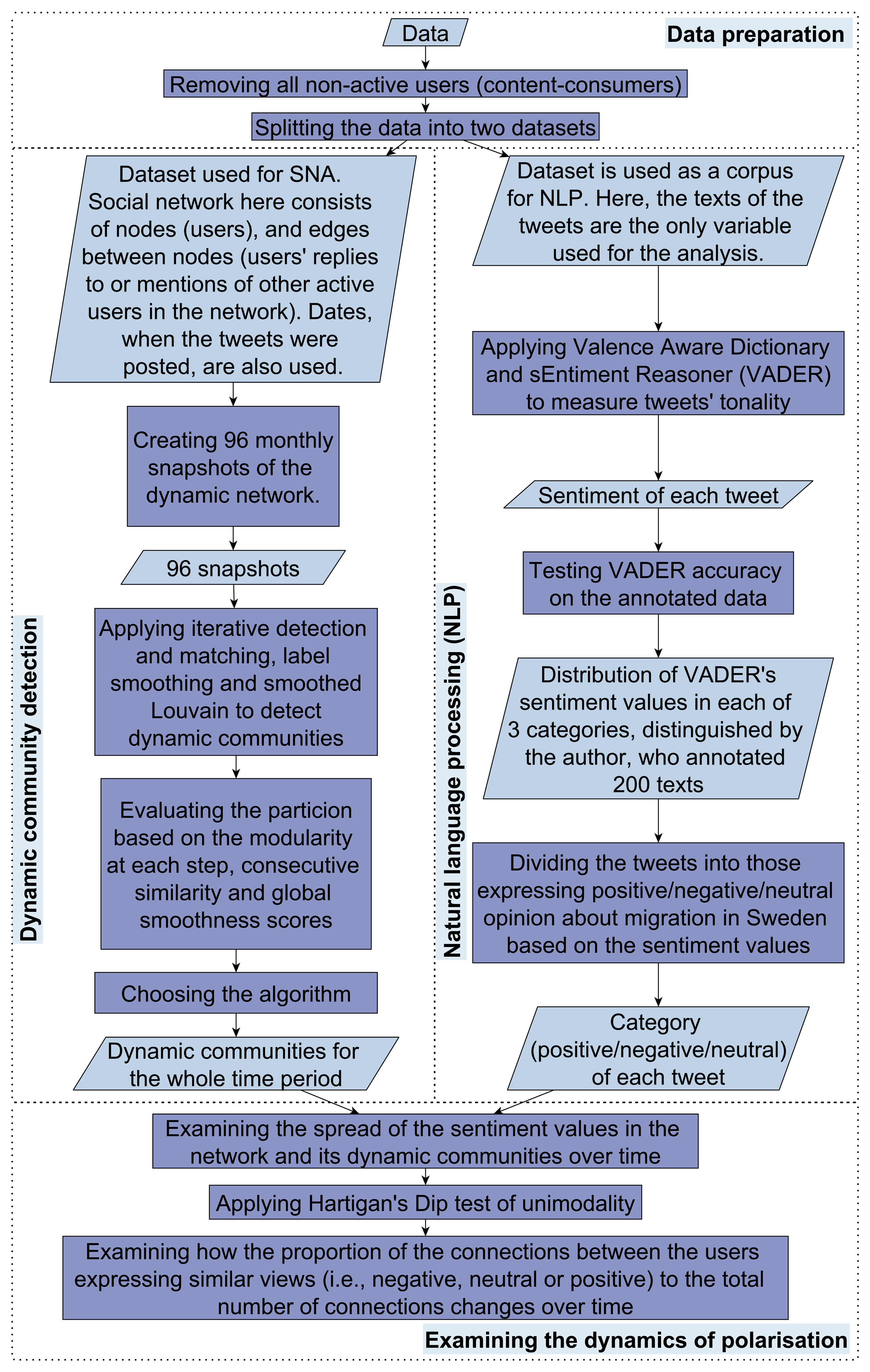

Supplement: S1 Fig — (TIF) [file pone.0262992.s001.tif]

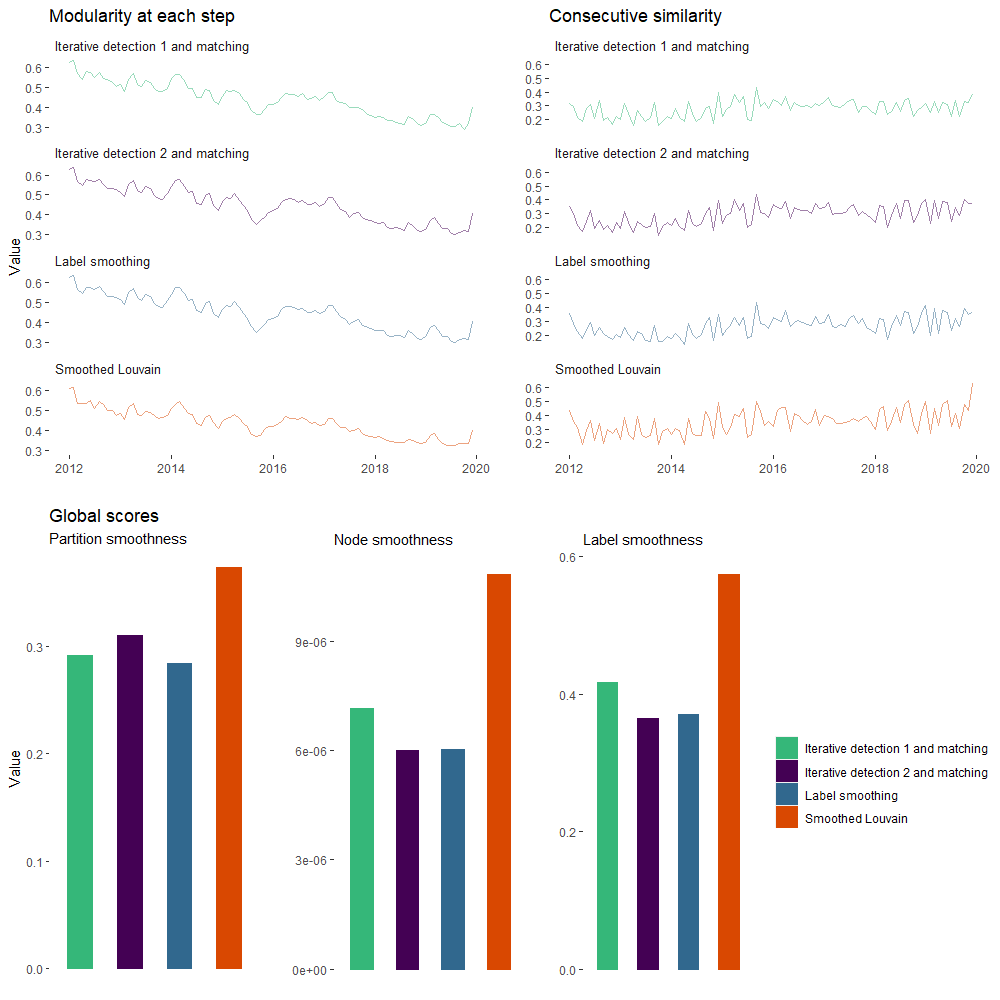

Supplement: S2 Fig — The Fig on the top left represents modularity at each step (X-axis) if one of the algorithms (Y-axis) is applied. The Fig on the top right represents consecutive similarity (X-axis) if one of the algorithms (Y-axis) is applied. The Fig on the bottom is the comparison of the algorithms depending on the global smoothness scores (Y-axis): from left to right, those are the average value of partition smoothness, node smoothness and label smoothness (X-axis). The figures evaluate the partition of the dynamic network into communities as the result of applying iterative detection and matching [98], label smoothing [99] and smoothed Louvain [100]. See the detailed description of the algorithms in [97]. (TIF) [file pone.0262992.s002.tif]

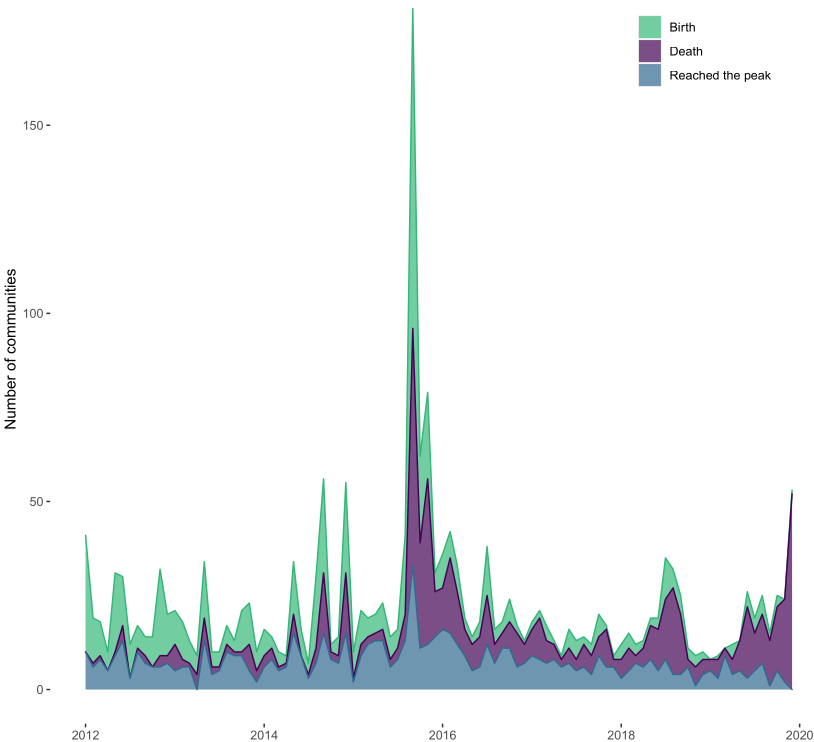

Supplement: S3 Fig — Y-axis shows the number of communities that emerged, died and reached the peak in terms of the number of users. The communities are received after applying iterative community detection using Clauset-Newman-Moore greedy modularity maximization and matching [97]. (TIF) [file pone.0262992.s003.tif]

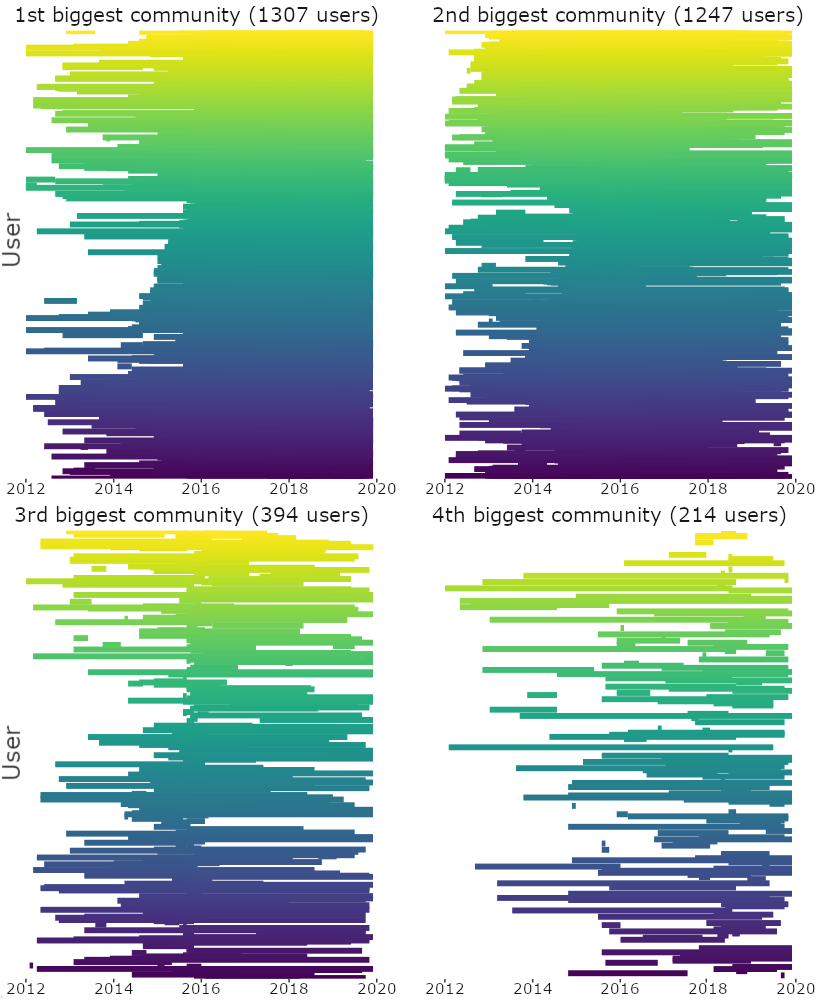

Supplement: S4 Fig — Y-axis shows the users activity over the examined time-period (X-axis). Each row represents the activity of one user. (TIF) [file pone.0262992.s004.tif]

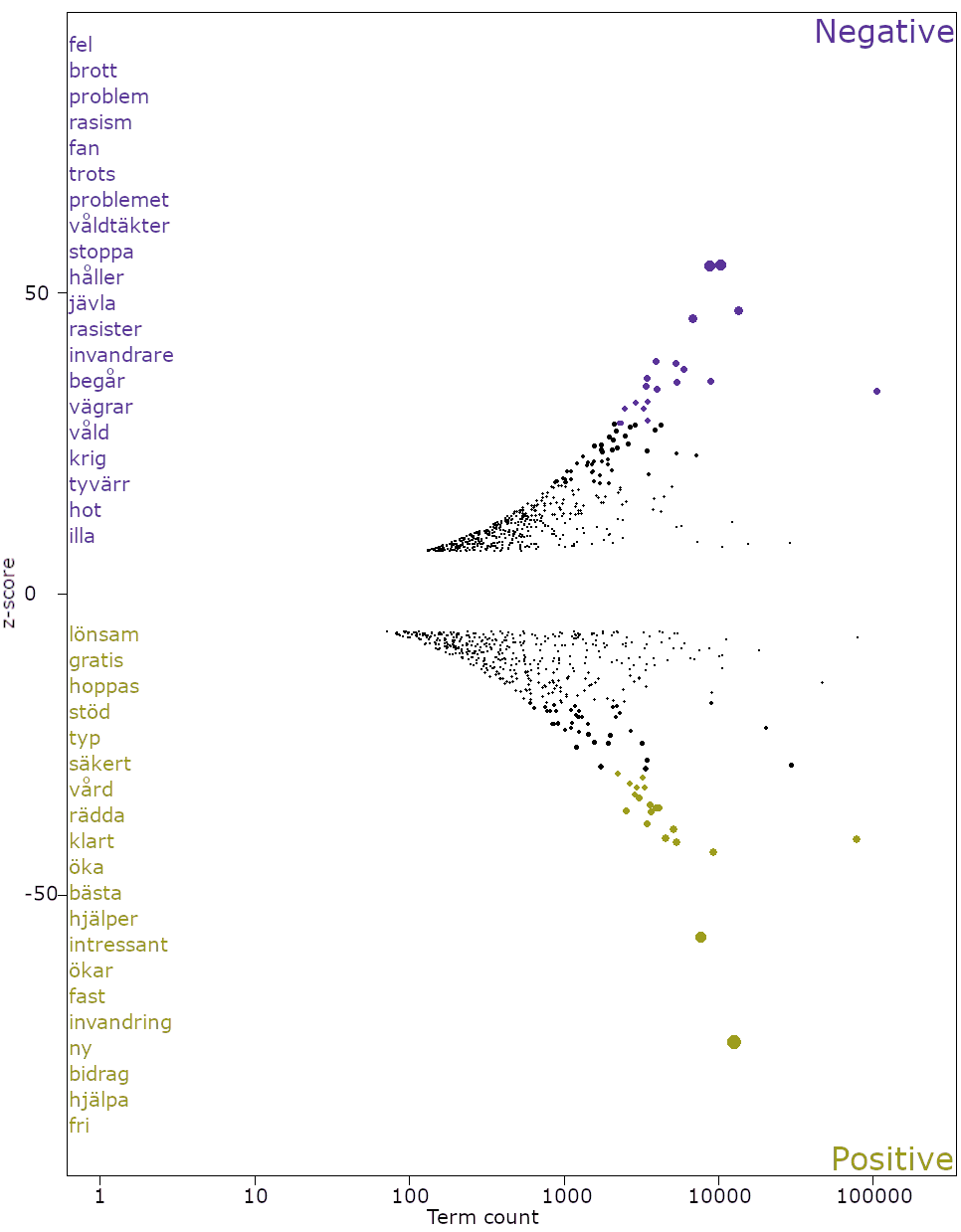

Supplement: S5 Fig — Term-category association (TCA) [105] analysis was applied to identify the words associated with the tweets expressing positive and negative opinions about migration in Sweden. N of tweets = 686 763. (TIF) [file pone.0262992.s005.tif]
