# Supplementary material for: Users’ polarisation in dynamic discussion networks: The case of refugee crisis in Sweden
Source: PLoS One. 2022 Feb 9;17(2):e0262992. doi: 10.1371/journal.pone.0262992 (PMC8827437; doi:10.1371/journal.pone.0262992)
Supplement: S1 File — The document provides additional information on the data handling and methods used for the analysis and complements the main text of the manuscript. (ZIP) [file pone.0262992.s006.zip › S1 File.pdf]

Supplementary information for:
Users' polarisation in dynamic discussion networks: the case of refugee crisis in Sweden

Elizaveta Kopacheva[1◑*], Victoria Yantseva[2◑**],

1 Department of Political Science & Centre for Data Intensive Sciences and Applications (DISA), Linnaeus University, Växjö, Sweden
2 Department of Social Studies & Centre for Data Intensive Sciences and Applications (DISA), Linnaeus University, Växjö, Sweden

◑The authors contributed equally and are listed in alphabetical order
* elizaveta.kopacheva@lnu.se
** victoria.yantseva@lnu.se

Introduction

This document provides additional information on the data handling and methods used for the analysis and complements the main text of the manuscript. We included details on: (1) data handling and (2) methods.

Data handling

The information was retrieved via the official Twitter full-archive search using the tools of *rtweet* package [1] in the R computing environment [2]. The query included the following terms: refugee(-s), migrant (-s), immigrant (-s), asylum seeker (-s), newcomers, immigration and migration (flykting (-ar, -na), invandrare (-na), migrant (-er), asylsökande, nyanlända, invandring (-en), migration (-en)). Within the study, we analysed batch data as opposed to real-time data. Thus, 1 198 985 Twitter messages posted within the time-period of the 1st of January 2012 to the 31st of December 2019 were collected and analysed. After retrieving information using the tools of *rtweet* package [1], we received a dataframe containing 90 variables providing information about users (such as user id, user's screen name, number of followers, etc.) and their tweets (text of the tweet, its length, information on if it is a reply to a post, date when the tweet was posted, etc.).

We treated the data of different types according to the purposes of the study, thus, we needed a network of users to analyse the development of user communities and in the meanwhile, the text of the messages was essential to track the changes in users' opinions in regard to migration in Sweden. In terms of this study, we also focused only on the content-creating users, i.e., users who posted more than 5 messages in a month. In that way, we managed to substantially reduce the noise in the data and remove network users that were outside of the dynamic communities. Due to the fact that the purpose of the study was to examine the dynamics of polarisation in online communities, keeping non-active users would not provide additional information to answer the research question.

Fig 1. The step-wise analysis of the retrieved data.

Methods

Once cleaning the data from non-active users (i.e., content-consumers), we applied social network analysis (SNA) and natural language processing (see Fig 1) to a number of variables providing information about the user's activity. Two sets of techniques were applied to the data independently from one another, however, at the later stage of the analysis, i.e., when examining the dynamics of polarisation, we combined the results of both steps (i.e, application of the social network analysis and natural language processing) to draw conclusions.

Dynamic community detection

Due to the constraints of the existing tools developed to detect dynamic communities (those constraints were elaborated upon in the manuscript), within this study, we approached the dynamic community detection by creating 96 monthly snapshots of the dynamic network and tracking the evolution of communities using and comparing the results of iterative detection and matching [3], label smoothing [4] and smoothed Louvain [5] (see the part of Fig 1 titled "Dynamic community detection"). We compared the performance of the algorithms by assessing the modularity at each step, consecutive similarity and global smoothness scores, such as the average value of partition smoothness, node smoothness and label smoothness [6]. The results of the assessment are presented by Fig 2.

Fig 2. The results of the dynamic partition evaluation. *Notes:* The Fig on the top left represents modularity at each step (X-axis) if one of the algorithms (Y-axis) is applied. The Fig on the top right represents consecutive similarity (X-axis) if one of the algorithms (Y-axis) is applied. The Fig on the bottom is the comparison of the algorithms depending on the global smoothness scores (Y-axis): from left to right, those are the average value of partition smoothness, node smoothness and label smoothness (X-axis). The figures evaluate the partition of the dynamic network into communities as the result of applying iterative detection and matching [3], label smoothing [4] and smoothed Louvain [5]. See the detailed description of the algorithms in [6].

As the result of community evolution tracking, we received the lists of users belonging to different dynamic communities as well as the information on when each community emerged, when it reached its peak in terms of the user count and when it disappeared. The following Fig 3 shows the dynamics of community emergence, death and reaching the peak.

Fig 3. Dynamic community birth, death and peak in terms of the number of users by month. *Notes:* Y-axis shows the number of communities that emerged, died and reached the peak in terms of the number of users. The communities are received after applying iterative community detection using Clauset-Newman-Moore greedy modularity maximization and matching [6].

Fig 3 shows that at the end of 2015 the largest number of communities appeared, died and reached the peak. The dynamics of the right side of the Fig 3, however, suggest that community cycles repeat the pre-crisis dynamics.

Moreover, examining user participation in the biggest dynamic communities, we can see that many users participate in the discussions since the beginning of the examined

time-period till 2020 (see Fig 4). This fact speaks against the Ship of Theseus effect [7].

Fig 4. User participation in Twitter discussions in 4 biggest dynamic communities. *Notes:* Y-axis shows the users activity over the examined time-period (X-axis). Each row represents the activity of one user.

Natural language processing

Simultaneously with detecting dynamic communities in the network of users, we processed the texts of the tweets posted by the active users to divide the tweets into those expressing positive, negative or neutral sentiments about immigration in Sweden.

To do so, we proceeded as follows in the part of Fig 1 titled "Natural language processing (NLP)".

Firstly, we used a Swedish version of the Valence Aware Dictionary and sEntiment Reasoner (VADER)[8] to measure the tonality of the texts. Thus, we received the sentiment value of each tweet ranging from -1 (signifying the negative sentiment) to +1 (suggesting positive tonality).

Secondly, we tested the model on 200 manually annotated tweets divided into 3 categories, i.e., positive, negative and neural. That allowed us to not only measure the accuracy, precision and recall of the model but also to fine-tune the division of the sentiment values into three specified categories. We layered two approaches to do that. Thus, we calculated the initial border values using the estimated kernel cumulative distribution function (CDF), such as $1 - CDF_{neg+neu}(s_1) = CDF_{pos}(s_1)$ and $1 - CDF_{pos+neu}(s_2) = CDF_{neg}(s_2)$, where $CDF_{pos/neu/neg}$ is the cumulative distribution function of sentiment values in the category positive/negative or neutral and $s_{1/2}$ is the border sentiment value. After that, we compared the performance of the model using all variations of the border values in the intervals $[s_1 - 0.3; s_1 + 0.3]$ and $[s_2 - 0.3; s_2 + 0.3]$ maximising the accuracy of the model on the annotated data, the value of $y = (T_{pos} - F_{pos}) + (T_{neg} - F_{neg})$ (as suggested by [9]), where $T_{pos/neg}$ are the true positive or negative and $F_{pos/neg}$ are the false positive or negative values, and recall for all of the categories. The values closest to the initial (s_1 and s_2) and the ones that maximise accuracy, $y = (T_{pos} - F_{pos}) + (T_{neg} - F_{neg})$ and recall were chosen. We layered two approaches, for one thing, to compensate for the limitations related to a low volume of the annotated dataset. Secondly, the estimated kernel cumulative distribution function treats all categories equally, however, we aimed to increase the accuracy of identifying positive and negative tweets, in particular, thus, penalised misidentification of positive versus negative tweets. In our case, the layered technique performed better. Table 1 shows the confusion matrix when applying two different approaches. Here, we can see that while one negative tweet was identified as neutral when applying the layered approach, this tweet was identified as positive when using CDF.

Table 1. Confusion matrices comparing CDF-based classification with the layered approach.

	**Layered approach Predicted sentiment**			**CDF approach Predicted sentiment**		
**Actual sentiment**	negative	neutral	positive	negative	neutral	positive
negative	74	17	11	74	16	12
neutral	17	36	25	17	36	25
positive	2	4	14	2	4	14

Notes: N of annotated tweets = 200.

To check for robustness, we also, applied term-category association (TCA) analysis (see [10] for more information) and found the results to be consistent with the expectations. Fig 5 shows the top 20 words associated with the tweets expressing positive and negative opinions about migration in Sweden.

Fig 5. Top 20 words associated with the tweets expressing positive and negative opinions about migration in Sweden. *Notes:* Term-category association (TCA) [10] analysis was applied to identify the words associated with the tweets expressing positive and negative opinions about migration in Sweden. *N of tweets* = 686 763.

Examining the dynamics of polarisation

Once the dynamic network communities were detected and the tweets were divided into three groups of messages with positive, neutral and negative sentiment polarity, we proceeded with the final stage of the analysis, i.e., examining the dynamics of polarisation in the network and dynamic clusters (see part of Fig 1 titled "Examining the dynamics of polarisation"). Here, we examined the changes of the sentiment distribution over time and tested for bimodality. We also measured the homogeneity of the dynamic communities to test the third hypothesis. To do so, we computed the average sentiment of each user per month. If the user did not post anything within the month but was replied to or mentioned, we replaced the missing value with the last sentiment of the user within its timeline. These decisions may have affected the results of the analysis, however, are justified based on the logic of the social media network formation. In particular, if user A mentions or replies to user B and user B did not post anything at the particular month, user A's perception of user B is based on the previous activity of user B.

Additional resources

Access the Supplementary R/Python script used for the analysis via `https://elizabethkopacheva.github.io/CET-W2V/`.

References

1. Kearney MW. rtweet: Collecting and analyzing Twitter data. Journal of Open Source Software. 2019;4(42):1829. doi:10.21105/joss.01829.
2. R Core Team. R: A language and environment for statistical computing; 2020. Available from: `https://www.R-project.org/`.
3. Greene D, Doyle D, Cunningham P; Science Foundation Ireland. Tracking the evolution of communities in dynamic social networks. N Memon and R Alhajj (eds) 2010 International Conference on Advances in Social Network Analysis and Mining : ASONAM 2010 : proceedings. 2010;doi:10.1109/ASONAM.2010.17.
4. Falkowski T, Bartelheimer J, Spiliopoulou M. Mining and visualizing the evolution of subgroups in social networks. In: Proceedings of the 2006 IEEE/WIC/ACM International Conference on Web Intelligence. WI '06. USA: IEEE Computer Society; 2006. p. 52–58. Available from: `https://doi.org/10.1109/WI.2006.118`.

5. Aynaud T, Guillaume JL. Static community detection algorithms for evolving networks. In: WiOpt'10: Modeling and optimization in mobile, ad hoc, and wireless networks. Avignon, France; 2010. p. 508–514. Available from: https://hal.inria.fr/inria-00492058.

6. Cazabet R. Tnetwork - Network community library; 2021. Github. Available from: https://tnetwork.readthedocs.io/en/latest/reference/generated/tnetwork.DCD.iterative_match.html.

7. Cazabet R, Rossetti G. Challenges in community discovery on temporal networks. ArXiv. 2019;abs/1907.11435. doi:10.1007/978-3-030-23495-9_10.

8. Hutto CJ, Gilbert E. VADER: A parsimonious rule-based model for sentiment analysis of social media text. Proceedings of the 8th International Conference on Weblogs and Social Media, ICWSM 2014. 2015; p. 216–225.

9. Serviss JT, Gådin JR, Eriksson P, Folkersen L, Grandér D. ClusterSignificance: A bioconductor package facilitating statistical analysis of class cluster separations in dimensionality reduced data. Bioinformatics. 2017;33(19):3126–3128. doi:10.1093/bioinformatics/btx393.

10. Denny MJ. SpeedReader: High performance text analysis; 2018. Available from: https://github.com/matthewjdenny/SpeedReader.
